# Transcriptomic and metabolomics responses to elevated cell wall invertase activity during tomato fruit set

**DOI:** 10.1093/jxb/erx219

**Published:** 2017-07-20

**Authors:** Lei Ru, Sonia Osorio, Lu Wang, Alisdair R Fernie, John W Patrick, Yong-Ling Ruan

**Affiliations:** 1 School of Environmental and Life Sciences, University of Newcastle, Callaghan, NSW, Australia; 2 Australia-China Research Centre for Crop Improvement, University of Newcastle, Callaghan, NSW, Australia; 3 Max Planck Institute of Molecular Plant Physiology, Potsdam-Golm, Germany

**Keywords:** Cell wall invertase, fruit set, metabolome, ovary, *Solanum lycopersicum*, transcriptome

## Abstract

Fruit set is a developmental transition from ovaries to fruitlets that determines yield potential. Cell wall invertase (CWIN) is essential for fruit and seed set, but the underlying molecular basis remains elusive. We addressed this issue by using CWIN-elevated transgenic tomato, focusing on ovaries and fruitlets at 2 d before and after anthesis, respectively. RNAseq analyses revealed that ovaries and fruitlets exhibited remarkable differences in their transcriptomic responses to elevated CWIN activity. Ovaries 2 d before anthesis were far more responsive to elevated CWIN activity compared with the fruitlets. We identified several previously unknown pathways that were up-regulated by elevated CWIN activity during fruit set. The most notable of these were expression of genes for defence, ethylene synthesis and the cell cycle along with a large number of cell wall-related genes. By contrast, expression of photosynthetic, protein degradation and some receptor-like kinase genes were generally decreased as compared with the wild type ovaries. GC-MS analyses revealed that 22 out of 24 amino acids exhibited reduced levels in the RNAi ovaries as compared with that in the wild type, probably owing to a down-regulated expression of protein degradation genes. Overall, the data indicate that (i) ovaries are much more sensitive to metabolic intervention than fruitlets; (ii) high CWIN activity could promote fruit set by improving resistance against pathogens and altering cell cycle and cell wall synthesis.

## Teaser

Ovaries are transcriptionally much more sensitive to metabolic intervention than fruitlets and elevation of CWIN activity increased the expression of R and PR genes in ovaries for defence against pathogens.

## Introduction

Fruit set is a critical process determining yield potential of many crops. Most studies in fruit biology, however, have focused on mid-to-late stages of development, especially in relation to fruit expansion (Carrari and Fernie, 2006), sugar accumulation (Beauvoit *et al.*, 2014) and ripening (Alba *et al.*, 2005). Only a limited number of studies have explored the molecular regulation of fruit set, characterized by the transition from quiescent ovaries to rapidly growing fruitlets, the earliest stage of fruit development (Vriezen *et al.*, 2008; Wang *et al.*, 2009).

Cell wall invertase (CWIN) hydrolyses sucrose (Suc) to glucose (Glc) and fructose (Fru) in the apoplasm and has been shown to play important roles in the development of sink organs, including fruit and seed (Ruan *et al.*, 2012; Wang and Ruan 2012). A classic example is from the *miniature 1* mutant in maize, which exhibits a miniature seed phenotype due to a mutation in the *Mn1* gene encoding a CWIN, INCW2, leading to reduced CWIN activity and cell division (Cheng *et al.*, 1996). In rice, *GIF1*, encoding a CWIN, is a key regulator of grain filling. Overexpressing *GIF1*, driven by its native promoter, resulted in larger and heavier grains in the transgenic line (Wang *et al.*, 2008). Silencing of a CWIN gene, *Lin5*, in tomato led to fruit abortion (Zanor *et al.*, 2009), while elevated CWIN activity promoted fruit and seed development (Jin *et al.*, 2009; Liu *et al.*, 2016). The hexoses produced from CWIN-mediated hydrolysis of Suc unloaded to the ovary/fruitlet apoplasm could function both as signalling molecules to regulate development and as an energy source and building blocks for metabolism and growth. Ruan *et al.* (2012) proposed a model of sugar-mediated seed and fruit seed set, in which a Glc signal, produced by CWIN, is hypothesized to promote cell division and to suppress the programmed cell death pathway for successful fruit set. However, it remains unknown which molecular pathways underlie CWIN-mediated fruit set.

We aimed to identify which genes and molecular pathways are responsive to elevated CWIN activity during tomato fruit set, focusing on the ovary-to-fruit transition period of 2 d before anthesis (dba) ovaries to 2 d after anthesis (daa) fruitlets. To this end, *LIN5* and *SlINVINH1* are the only CWIN and CWIN inhibitor genes, respectively, expressed in tomato ovaries and fruits (Jin *et al.*, 2009; Palmer *et al.*, 2015). Silencing *SlINVINH1* elevated CWIN activity by ~ 35% in 2 dba ovaries and 2 daa fruitlets (Liu *et al.*, 2016). Unlike some ectopic expression studies, where misexpression of an introduced foreign gene often causes pleiotropic and detrimental effects on growth and development, no foreign CWIN genes are introduced in this case and the elevation of CWIN activity was derived entirely from the endogenous CWIN enzyme (Jin *et al.*, 2009). This provides a valuable opportunity to assess the role of the native CWIN in tomato fruit set without potential complications from misexpression of foreign CWIN genes.

We identified possible molecular pathways responsive to elevated CWIN activity by using transcriptomic and metabolomic approaches. The analyses revealed a number of novel findings including the following: (i) ovaries were much more responsive to elevated CWIN activity as compared with fruitlets, and (ii) elevated CWIN activity enhanced the expression of genes involved in pathogen resistance, ethylene synthesis, cell cycle and cell wall synthesis/remodelling, but reduced the expression of genes associated with photosynthesis and protein degradation as well as those encoding some receptor like kinases (RLKs). Overall, our data provide important insights into the molecular pathways by which CWIN regulates fruit set.

## Materials and methods

### Plant material

Transgenic tomato plants (*Solanum lycopersicum* XF-2) were used in which CWIN activity was elevated by RNAi silencing of its inhibitor gene, *SlINVINH1* (Jin *et al.*, 2009). The transgenic and WT plants were grown in a glasshouse under natural light, with day and night temperatures of 25 and 18 °C for 14 and 10 h, respectively. Plants were raised in 25-cm diameter (8 litres) pots filled with potting mix (1 part coarse sand, 1 part perlite and 1 part coir-peat), with one plant per pot. Standard Osmocote™/Osmocote high (K) potassium™ 1:1 slow release fertilizer (Scotts) was applied at a rate of 20 g per pot, supplemented with a weekly liquid fertilizer regime of Jurox Wuxal Liquid Foliar Nutrient Fertilizer™ at a diluted concentration of 4 ml l^−1^. Pots were maintained at field capacity through being watered twice a day, each of 3 min duration, by an automated drip irrigation system.

Tomato fruit age was determined by tagging flowers on the day of anthesis. To ensure synchronous pollination, each flower of interest was manually pollinated using a battery-powered vibrator at 10:00–11:00 h on the day of anthesis. This technique has proven to be highly effective in releasing sufficient pollen grains for synchronous pollination and fruit set (e.g. Palmer *et al.* 2015; Liu *et al.*, 2016). Ovaries and fruitlets at 2 dba and 2 daa, respectively, were harvested and frozen in liquid nitrogen immediately and then stored at −80 °C until use.

### RNA isolation and Illumina sequencing

Total RNA was extracted from 2 dba ovaries and 2 daa fruitlets of WT and *SlINVINH1*-RNAi plants using Qiagen RNA Plant Mini Kits. Genomic DNA was removed by using DNase (Promega). A 5 μg subsample of total RNA from each of 12 RNA extracts (2 stages×2 genotypes×3 biological replicates) were sent to the Australia Genome Research Facility Ltd (AGRF) in Melbourne, Australia. The Illumina Hiseq-2000 platform was used to produce 100 bp single-end runs.

Total RNA quality was determined using an Agilent 2100 Bioanalyzer and quality of the sequenced raw reads were assessed by FastQC (http://www.bioinformatics.babraham.ac.uk/projects/fastqc/).

### RNA-seq data analysis

The raw reads were screened for the presence of any adaptor/overrepresented sequences and ambiguous characters and clipped where required. TopHat 2 (version 2.0.9), which employed bowtie2 (version 2.1.0.0) and Samtools (version 0.1.17.0), was used to align RNA-Seq reads to the tomato genome reference (ITAG release 2.3, ftp://ftp.solgenomics.net/tomato_genome/annotation/ITAG2.3_release/), with the option ‘ITAG2.3_gene_models.gff3’ being provided to supply TopHat with a set of gene model annotations of tomato. HTSeq (version 0.5.4p2) was then used to quantify the mRNA levels by generating the raw count data for each sample. Based on the raw count data, edgeR (edgeR version 3.2.4, Bioconductor version 2.12, R version 3.0.0) was adopted to perform the differential expression analysis. We conducted this analysis for four different categories: (i) ‘WT fruitlets’ *vs* ‘WT ovaries’; (ii) ‘*SlINVINH1*-RNAi fruitlets’ *vs* ‘*SlINVINH1*-RNAi ovaries’; (iii) ‘*SlINVINH1*-RNAi ovaries’ *vs* ‘WT ovaries’; and (iv) ‘*SlINVINH1*-RNAi fruitlets’ *vs* ‘WT fruitlets’. Differentially expressed genes (DEGs) in each category were further compared to identify the overlapped and category-specific gene sets using custom PERL scripts. DEGs were defined by satisfying the following criteria: (i) reads per kilobase of transcript per million mapped reads (RPKM) is greater than 0.45 (Zhang *et al.*, 2015); (ii) false discovery rate (FDR) <0.05; and (iii) log_2_FC is greater than 0.5 or less than −0.5 (Wang *et al.*, 2009). MAPMAN (Thimm *et al.*, 2004) was used to identify functional categories of the DEGs.

### Metabolome analysis

Samples of ovaries (2 dba) and fruitlets (2 daa) were freeze-dried and a 5 g (dry weight) subsample for each of the 12 samples (2 stages×2 genotypes×3 biological replicates) was sent to Max-Planck-Institut für Molekulare Pflanzenphysiologie, Potsdam-Golm, Germany for primary metabolite profiling analysis. Metabolite extraction, sample derivatization, standard addition and sample injection for gas chromatography–mass spectrometry (GC-MS) analyses were performed as described by Osorio *et al.* (2012). The mass spectra were cross-referenced with those in the Golm Metabolome Database (http://gmd.mpimp-golm.mpg.de/) (Kopka *et al.*, 2004).

### Principal component analyses

CLC Genomics Workbench (9.0) was used for principal component analysis (PCA) of RNA-seq and metabolomic data sets.

### qRT-PCR

RNA extraction and qRT-PCR were carried out as described previously (Palmer *et al.*, 2015). *SlCAC* and *SlTIP41* were used as reference genes as the expression levels of these genes were most stable from the ovary-to-fruitlet stages in both RNAi and WT plants. Primers used for qRT-PCR were list in Supplementary Table S1 at *JXB* online.

## Results

### Identification of differentially expressed genes during fruit set in *SlINVINH1*-RNAi and WT plants

As the first step, we confirmed that the CWIN activity was elevated in ovaries and fruitlets of the *SlINVINH1*-RNAi plant. Enzyme assays revealed that CWIN activity was indeed significantly increased in the 2 dba ovaries and 2 daa fruitlets compared with that in WT plants (see Supplementary Fig. S1), consistent with our recent report (Liu *et al.*, 2016). Importantly, the activities of vacuolar invertase (VIN) and cytosolic invertase (CIN) were not affected in the transgenic plants (Supplementary Fig. S1), demonstrating the specific inhibitory effect of SlINVINH1 against CWIN as shown previously (Jin *et al.*, 2009). Following this validation, we conducted RNAseq analyses to discover which genes or gene networks responded to the elevated CWIN activity in the RNAi plants.

**Fig. 1.**
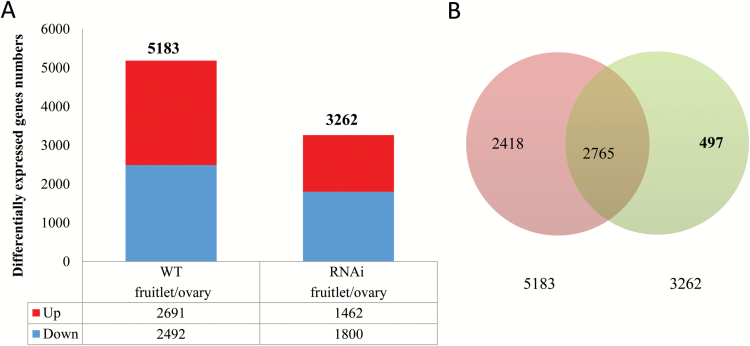
Differentially expressed genes during fruit set in wild type (WT) and *SlINVINH1*-RNAi tomato. (A) Numbers of differentially expressed genes during WT and *SlINVINH1*-RNAi fruit set (2 daa fruitlet *vs* 2 dba ovary). The upper part of each bar represents up-regulated and the lower part down-regulated genes. Total numbers of differentially expressed genes are listed on top of the bar for each group. Numbers of down- or up-regulated expressed genes are listed at the bottom of the figure. (B) A Venn diagram showing differentially expressed genes that are unique to fruit set of *SlINVINH1*-RNAi plants. There were 2765 (overlapped area) shared by both genotypes during fruit set and 497 differentially expressed genes were specifically responsive to elevated CWIN activity in the *SlINVINH1*-RNAi plants. (This figure is available in colour at *JXB* online.)

Among the total 34 727 genes in the tomato genome, our RNAseq analysis indicated that about 58% of them were expressed in the 2 dba ovaries and 2 daa fruitlets with little difference between the two developmental stages or between RNAi and WT plants. We then identified differentially expressed genes (DEGs) at fruit set of WT and *SlINVINH1*-RNA interference (RNAi) plants. In total, 5183 and 3262 DEGs were detected in WT and *SlINVINH1*-RNAi plants, respectively, during the ovary-to-fruit transition (Fig. 1A, Supplementary Tables S2 and S3). These represent 15.0% and 9.4% of the genes in the tomato genome, respectively. It is intriguing that DEG numbers were much less in *SlINVINH1*-RNAi compared with WT plants during fruit set (Fig. 1A). The number of DEGs (5183) detected during WT fruit set was four times higher than those reported by Wang *et al.*, (2009) who identified 1298 DEGs during tomato fruit set from 2 dba to 4 daa. These authors used a microarray approach that does not provide genome-wide coverage. The RNA sequencing applied in our study overcame this obstacle, leading to the detection of many more expressed genes.

Functional categorization, using MAPMAN, of the DEGs (5183) in the WT samples revealed that major groups exhibiting changes during fruit set belong to categories for protein (BIN 29), RNA (BIN 27), and transport (BIN 34). The protein (BIN 29) category consisted of 14.7% of the total DEGs during WT fruit set (Table 1). The protein category (BIN 29) contains protein synthesis (BIN 29.2), protein post-translational modification (BIN 29.4), and protein degradation (BIN 29.5) (Thimm *et al.*, 2004). RNA (BIN 27) was the second major group, containing 12.9% of the total DEGs in WT fruit set (Table 1) and can be subdivided into RNA processing (BIN 27.1), RNA translation (BIN 27.2), regulation of transcription (transcription factor, BIN 27.3), and RNA binding (BIN 27.4) (Thimm *et al.*, 2004). A similar grouping of functional categories to WT fruit set was found during fruit set of the *SlINVINH1*-RNAi line, but with a lower number of DEGs in each of these functional groups. The top two functional groups were protein (BIN 29) and RNA (BIN 27). The ranking of other functional groups was slightly altered in the *SlINVINH1*-RNAi compared with WT fruit set (Table 1). The large number of genes involved in protein (BIN 29) and RNA (BIN 27) in both genotypes indicates that fruit set is a process undergoing rapid cell development with intensive protein and RNA synthesis, regulation and turnover.

**Table 1.** *Functional classification of differentially expressed genes during fruit set in wild type (WT) and* SlINVINH1*-RNAi plants (CWIN activity up-regulated*) Differentially expressed genes were ascribed to 36 bins in MAPMAN (Thimm *et al.*, 2004). Only the major functional groups are listed. The order was made based on the number of differentially expressed genes in a given functional category (BIN), excluding the categories of ‘Others’ and ‘Unknown’.

Functional category	WT fruit set (fruitlet/ovary)			*SlINVINH1*-RNAi fruit set (fruitlet/ovary)		
	No.	Percentage	Rank	No.	Percentage	Rank
Protein (BIN 29)	761	14.7	1	506	15.5	1
RNA (BIN 27)	671	12.9	2	394	12.1	2
Transport (BIN 34)	297	5.7	3	187	5.7	3
Signalling (BIN 30)	270	5.2	4	149	4.6	5
Hormone metabolism (BIN 17)	179	3.8	5	137	4.7	4
Cell (BIN 31)	190	3.7	6	87	2.7	9
Stress (BIN 20)	186	3.6	7	138	4.2	6
Cell wall (BIN 10)	146	2.8	8	101	3.1	8
Development (BIN 33)	144	2.8	9	115	3.5	7
Lipid development (BIN 11)	131	2.5	10	81	2.5	11
Secondary metabolism (BIN 16)	114	2.2	11	84	2.6	10
DNA (BIN 28)	102	2.0	12	46	1.4	13
Amino acid metabolism (BIN 13)	70	1.4	13	45	1.4	14
Photosynthesis (BIN 1)	52	1.0	14	49	1.5	12
Sucrose and starch metabolism (BIN 2)	23	0.4	15	14	0.4	15
Others and Unknown	1808	34.9	—	1136	34.8	—
Total	5183			3262		

### Photosynthesis-related genes were predominately down-regulated during fruit set among the 497 DEGs unique to elevation of CWIN activity

To unravel the candidate genes that are regulated by the elevation of CWIN activity during fruit set process, we prepared a Venn diagram to show how many DEGs are unique to fruit set of S*lINVINH1*-RNAi plants. This analysis revealed that the majority of DEGs (2765) were common to both genotypes during fruit set, with only 497 DEGs uniquely responsive to elevated CWIN activity (Fig. 1B and Supplementary Table S4).

Of particular note, an interesting expression pattern of photosynthesis-related genes was observed among the 497 DEGs. With the exception for the gene encoding rubisco activase, which was up-regulated, expression levels of the other 13 of 14 DEGs encoding photosynthesis proteins were decreased in 2 daa fruitlets compared with 2 dba ovaries of *SlINVINH1*-RNAi plants (Table 2). These photosynthesis-related genes encode proteins functioning in different photosynthetic processes. Among them, nine transcripts encode proteins for the light reaction, one for photorespiration and four for the Calvin–Benson cycle. For those responsible for the light reactions, three transcripts encode chlorophyll *a*/*b* binding proteins (Table 2).

**Table 2.** *Elevation of CWIN activity in the* SlINVINH1*-RNAi plants suppressed the expression of photosynthesis-related genes during fruit set compared with that in the wild type tomato plants*

Gene locus	Annotation	Log _ 2 _ FC
Light reaction		
solyc08g067320.1.1	Chlorophyll *a*/*b* binding protein	−1.34
solyc05g056050.2.1	Chlorophyll *a*/*b* binding protein	−1.20
solyc08g067330.1.1	Chlorophyll *a*/*b* binding protein	−1.30
solyc02g065400.2.1	Oxygen-evolving enhancer protein	−0.55
solyc10g005050.2.1	Thylakoid membrane phosphorprotein	−0.77
solyc12g044280.1.1	Photosystem I reaction, subunit VI	−1.59
solyc10g075160.1.1	Ferredoxin I	−0.88
solyc05g026550.2.1	NADH dehydrogenase	−1.01
solyc08g080050.2.1	PGR5-like protein 1A	−0.59
Photorespiration		
solyc06g061070.2.1	Glycine cleavage system H	−0.66
Calvin cycle		
solyc06g009630.1.1	CP12	−0.52
solyc10g018300.1.1	Transketolase 1	−0.82
solyc03g117850.2.1	Rubisco activase	−0.54
solyc09g011080.2.1	Rubisco activase 1	0.79

**Fig. 2.**
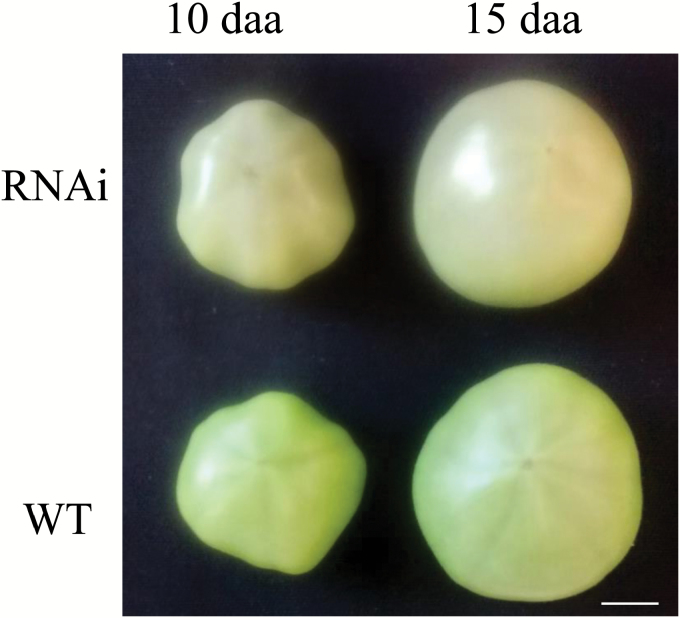
Tomato fruits of *SlINVINH1*-RNAi (with up-regulated CWIN activity) were a lighter green colour compared with those of wild type (WT) fruits at 10 and 15 d after anthesis (daa). Representative images are presented. Scale bar: 1 cm.

Consistent with the repression of a large number of photosynthesis genes, developing fruits of the transgenic plants were paler green at 10 and 15 daa compared with WT fruits (Fig. 2) although such a phenotype was not apparent for 2 dba ovaries or 2 daa fruitlets.

### Ovaries were transcriptionally much more sensitive to elevated CWIN activity than fruitlets

**Fig. 3.**
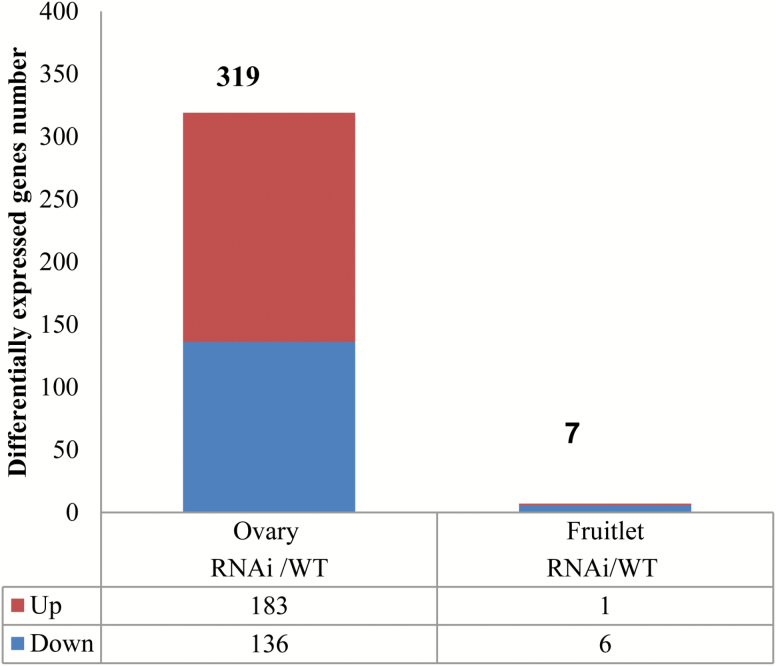
Differentially expressed genes in 2 daa ovaries and 2 daa fruitlets of *SlINVINH1*-RNAi (CWIN activity up-regulated) compared with those of wild type (WT) plants. The upper part of the bars represents up-regulated and the lower part down-regulated expressed genes. Total number of differentially expressed genes is listed above the bar for each group. Numbers of down- or up-regulated expressed genes are listed at the bottom of the figure. (This figure is available in colour at *JXB* online.)

We then compared the impact of elevated CWIN activity on the transcriptomes of 2 dba ovaries and 2 daa fruitlets between WT and *SlINVINH1*-RNAi plants. The response of ovaries and fruitlets to elevated CWIN activity was remarkably different. There were only seven DEGs found in 2 daa fruitlets between the two genotypes. However, surprisingly there were 319 DEGs observed in 2 dba ovaries between the two genotypes (Fig. 3). These data show that 2 dba ovaries were much more responsive to elevated CWIN activity than 2 daa fruitlets at the gene transcript level.

Among the seven DEGs in 2 daa fruitlets (Fig. 3), four transcripts belong to categories encoding proteins functioning in the cell wall, signalling, biotic stress, and lipid metabolism, with a further three transcripts encoding proteins of unknown function (Table 3). The four DEGs of known function encode a pectin esterase, a leucine-rich repeat (LRR) receptor-like serine/threonine-protein kinase, a resistance (R) protein containing a nucleotide binding site and leucine-rich repeat (NBS-IRR) domains, and cyclopropane-fatty-acyl-phospholipid synthase. Expression levels of six DEGs decreased in 2 daa fruitlets of *SlINVINH1*-RNAi compared with WT fruitlets, except for transcript levels of a gene encoding an R protein which increased in 2 daa fruitlets of *SlINVINH1*-RNAi compared with WT fruitlets (Table 3).

**Table 3.** *Differentially expressed genes in 2 daa fruitlets in response to elevated CWIN activity of* SlINVINH1*-RNAi compared with wild type fruitlets* LRR: leucine-rich repeat; NBS-IRR: nucleotide binding site-leucine-rich repeat domains.

Functional category	Gene locus	Annotation	Log _ 2 _ FC
Cell wall	solyc01g091050.2	Pectin esterase	−1.53
Signalling	solyc06g008270.2	LRR receptor-like serine/threonine-protein kinase	−1.89
Biotic stress	solyc02g032650.2	Resistance protein, NBS-IRR	1.50
Lipid metabolism	solyc09g090500.2	Cyclopropane-fatty-acyl-phospholipid synthase	−2.41
Unknown	solyc12g062200.1	Unknown protein	−5.91
Unknown	solyc07g007040.2	Zinc finger CCCH type with G patch	−1.24
Not assigned	solyc07g062500.2	Cytochrome P450	−1.11

For the 319 DEGs detected in the genotypic comparison of 2 dba ovaries (see Supplementary Table S5), a functional categorization analysis revealed that DEGs encoded proteins primarily functioning in protein (BIN 29), hormone (BIN 17), RNA (BIN 27), stress (BIN 20), signalling (BIN 30), and cell wall (BIN 10) (Table 4). Since ovaries, not fruitlets, were the primary organ responsive to elevated CWIN activity in the *SlINVINH1*-RNAi plants, we focused our subsequent analyses on 2 dba ovaries in order to gain further insights into the DEGs regulated by increases in CWIN activity.

**Table 4.** *Functional classification of differentially expressed genes in 2 dba ovaries of* SlINVINH1*-RNAi compared with those of wild type (WT) plants* Differentially expressed genes were ascribed to 36 bins in MAPMAN (Thimm *et al.*, 2004). Some genes might be allocated to multiple different bins.

Functional category	Ovary (*SlINVINH1*-RNAi/ WT)	
	No. of genes	Percentage
Protein (BIN 29)	37	11.6
Hormone metabolism (BIN 17)	23	7.2
RNA (BIN 27)	20	6.3
Stress (BIN 20)	18	5.6
Signalling (BIN 30)	14	4.4
Cell wall (BIN 10)	13	4.1
Development (BIN 33)	11	3.5
Transport (BIN 34)	11	3.5
Cell (BIN 31)	9	2.8
Secondary metabolism (BIN 16)	7	2.2
Lipid metabolism (BIN 11)	5	1.5
DNA (BIN 28)	4	1.3
Sucrose and starch metabolism (BIN 2)	3	0.9
Photosynthesis (BIN 1)	2	0.6
Others^*a*^	10	3.1
Unknown (BIN 35 and BIN 26)	147	46.1
Total	319	

^*a*^Others include BIN 6 (gluconeogenesis/ glycosylate cycle), BIN 8 (TCA), BIN13 (amino acid metabolism), BIN 19 (tetrapyrrole synthesis), BIN 21 (redox), BIN 22 (polyamine metabolism), and BIN23 (nucleotide metabolism).

### Protein degradation-related genes were largely repressed in ovaries of *SlINVINH1*-RNAi plants in response to elevated CWIN activity

We detected 24 DEGs involved in protein degradation in ovaries of *SlINVINH1*-RNAi compared with WT plants (Table 5). These DEGs encode various proteases including subtilases, members of the subtilisin-like serine protease family (Siezen and Leunissen, 1997). All six DEGs encoding subtilisin-like proteases exhibited decreased transcript levels in ovaries of *SlINVINH1*-RNAi compared with WT plants. However, DEGs encoding cysteine protease, aspartate protease, and serine protease proteins, as well as those involved in the ubiquitin–proteasome pathways, exhibited either up- or down- regulation (Table 5). Overall ~70% (16 out of 24) of DEGs encoding proteins functioning in protein degradation exhibited a decreased expression in ovaries of *SlINVINH1*-RNAi compared with those of WT plants, indicating that protein degradation may be repressed in ovaries of *SlINVINH1*-RNAi compared with WT ovaries (Table 5).

**Table 5.** Differentially expressed genes in ovaries of *SlINVINH1*-RNAi compared with those of wild type plants

Functional category	Gene locus	Annotation	Log _ 2 _ FC
**Protein**			
Protein degradation	solyc09g083120.2.1	Peptidase S9	−1.03
	solyc09g083130.2.1	Peptidase S9	−0.61
	solyc05g052130.2.1	Metacaspase	−2.11
Subtilases	solyc04g078740.2.1	Subtilisin-like protease	−0.92
	solyc02g092670.1.1	Subtilisin-like protease	−0.66
	solyc12g088760.1.1	Subtilisin-like protease	−0.90
	solyc01g087740.1.1	Subtilisin-like protease	−0.89
	solyc01g087780.2.1	Subtilisin-like protease	−1.00
	solyc01g087790.2.1	Subtilisin-like protease	−1.35
Cysteine protease	solyc07g041920.2.1	Cathepsin L-like cysteine proteinase	0.72
Aspartate protease	solyc02g032940.2.1	Aspartic proteinase	−0.50
	solyc03g005280.2.1	Aspartic proteinase 2	0.84
	solyc03g058400.2.1	Aspartyl protease family	0.81
Serine protease	solyc02g082720.2.1	Serine protease	1.24
	solyc01g087970.2.1	Serine carboxypeptidase 1	−1.30
	solyc03g026080.2.1	Rhomboid family protein	−1.28
	solyc05g050770.2.1	Serine carboxypeptidase 1	−0.91
Ubiquitin.E2	solyc06g070980.2.1	Ubiquitin-conjugating enzyme E2 2	0.58
Ubiquitin.E3.RING	solyc12g015800.1.1	RING finger family protein	−0.82
	solyc10g008410.1.1	RING finger protein 5	1.05
	solyc07g020870.1.1	U-box domain-containing protein	0.75
	solyc12g088360.1.1	U-box domain-containing protein 4	−0.69
	solyc09g008430.2.1	CHY zinc finger family protein expressed	−0.97
Ubiquitin. proteasome	solyc10g054040.1.1	26S protease regulatory subunit	2.43
**Ethylene**			
Ethylene synthesis	solyc01g067620.2.1	ACC oxidase	1.22
	solyc02g071500.2.1	ACC oxidase 1	0.67
	solyc04g009850.2.1	ACC oxidase-like	2.52
	solyc04g009860.2.1	ACC oxidase-like	1.16
	solyc06g068270.2.1	ACC oxidase 1	−1.30
Ethylene signal transduction	solyc11g011740.1.1	ERF2	2.83
AP2/EREBP family	solyc03g114440.1.1	ERF3 (PR transcriptional factor)	2.26
	solyc07g054220.1.1	ERF2a (PR transcriptional factor)	0.78
**R protein**			
	solyc04g012010.2.1	CC-NBS-IRR	1.30
	solyc02g070730.1.1	CC-NBS-IRR	1.34
	solyc07g005770.2.1	CC-NBS-lRR	−0.80
	solyc07g039410.2.1	NBS-IRR	0.82
	solyc02g032650.2.1	NBS-IRR	0.76
	solyc01g008800.1.1	TIR-NBS-IRR	1.19
**PR protein**			
	solyc02g082920.2.1	Endochitinase	0.74
	solyc10g074440.1.1	Endochitinase	−1.97
**Cell wall**			
Cell wall synthase	solyc07g051820.2.1	Cellulose synthase-like	1.29
	solyc12g015770.1.1	Cellulose synthase-like	0.54
Cell wall degradation	solyc05g005080.2.1	Endo-1,4-β-glucanase	−0.51
	solyc05g052530.1.1	Endoglucanase 1	−1.07
	solyc11g044910.1.1	β-Xylosidase 1	−0.76
	solyc12g013770.1.1	Mannan endo-1,4-β-mannosidase	−1.20
Cell wall modification	solyc06g049050.2.1	Expansin	−1.00
	solyc02g091920.2.1	Xyloglucan endotransglucosylase/hydrolase 2	−2.02
	solyc05g047590.2.1	Pectinesterase	1.37
	solyc08g075020.2.1	Pectinacetylesterase like protein	−0.71
	solyc08g074950.1.1	Pectinacetylesterase	−0.87
	solyc08g074990.1.1	Pectinacetylesterase	−1.26
	solyc08g014380.1.1	Pectinacetylesterase	0.74
**Signalling**			
Receptor like kinase	solyc08g016210.2.1	LRR receptor-like serine/threonine-protein	−1.12
	solyc01g005760.2.1	LRR receptor-like serine/threonine-protein kinase	−1.19
	solyc01g005870.1.1	LRR receptor-like serine/threonine-protein kinase	−0.78
	solyc01g005720.2.1	LRR receptor-like serine/threonine-protein kinase	−1.46
	solyc04g008400.1.1	Serine/threonine-protein kinase receptor	−1.44
	solyc04g077270.2.1	Serine/threonine kinase receptor	−2.63
	solyc04g074000.2.1	Receptor like kinase	−2.29
	solyc02g071800.2.1	Receptor like kinase	−0.55
	solyc05g056370.2.1	Receptor like kinase	−0.50
	solyc11g011880.1.1	Receptor like kinase	0.55
	solyc12g008500.1.1	Receptor like kinase	1.22
Cytoplasmic kinase	solyc05g056370.2.1	Receptor-like kinase	−0.50
	solyc10g005300.2.1	Serine/threonine protein kinase	−1.72
Small GTPase signal	solyc02g077400.2.1	Small GTPase (ROP)	−0.91
**Cell cycle**			
	solyc04g081650.2.1	Cyclin B2	0.55
	solyc04g081660.2.1	Cyclin B2	0.53

### Expression of hormone-related genes in ovaries of *SlINVINH1*-RNAi plants in response to elevated CWIN activity

Considering the important roles hormones play in fruit set (de Jong *et al.*, 2009), and sugar-hormone interactions (Leon and Sheen, 2003; Kumar *et al.*, 2014), we closely examined hormone-related transcripts.

**Fig. 4.**
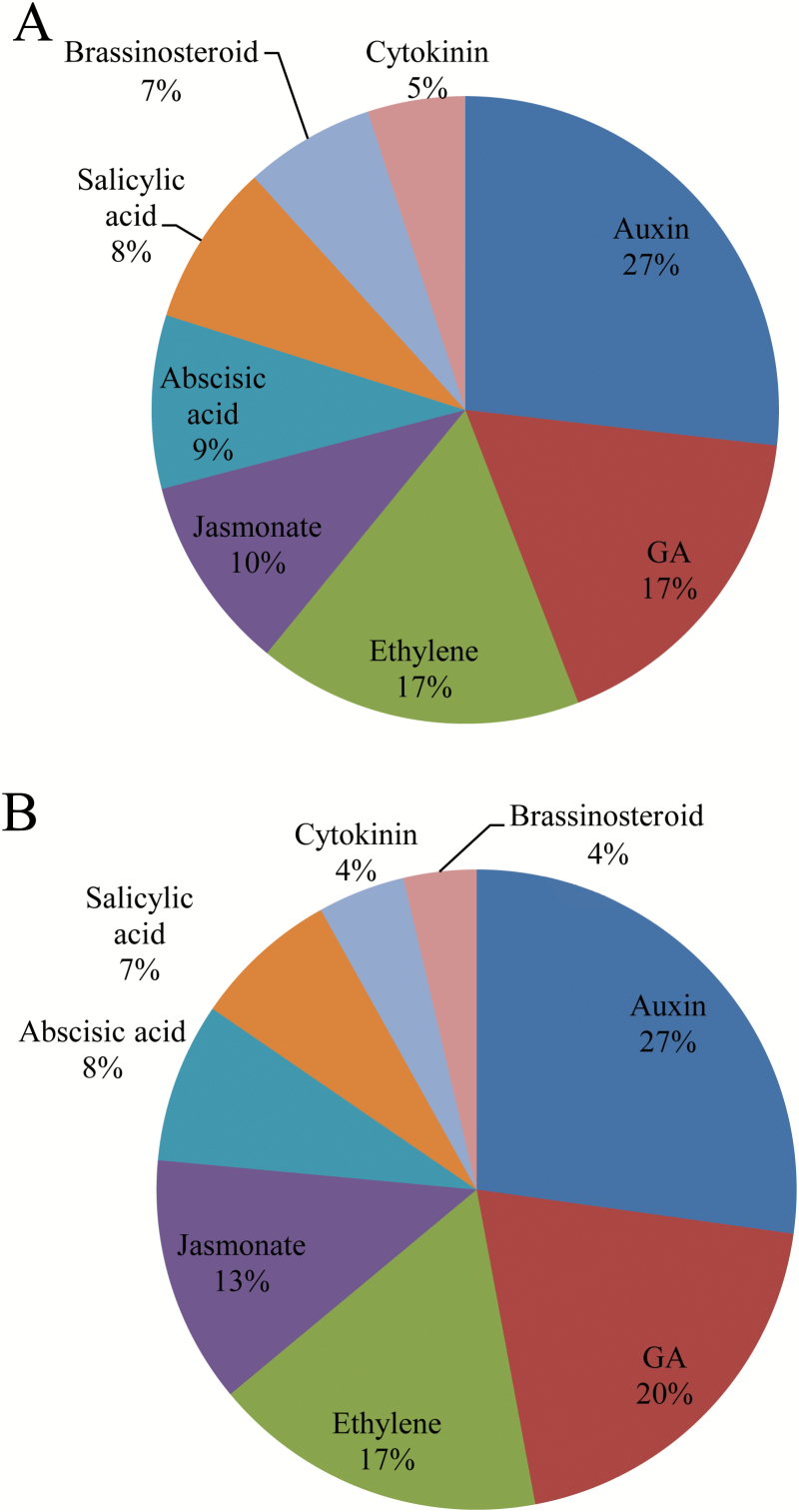
Percentages of expressed genes involved in hormone metabolism during fruit set. The numbers of differentially expressed genes involved in each hormone class expressed as percentages of the total numbers differentially expressed genes involved in hormone metabolism in fruit set in wild type plants (A) and *SlINVINH1*-RNAi plants (B). (This figure is available in colour at *JXB* online.)

During fruit set in WT plants, genes involved in auxin, GA, and ethylene metabolism and signalling are among the top three ‘hormones’ gene classes, representing 27%, 17%, and 17% of the total hormone-related genes respectively (Fig. 4). A similar expression profile was found in the *SlINVINH1*-RNAi plants, with genes involved in ethylene synthesis and signalling ranked the third most important hormone class in terms of DEG numbers (Fig. 4). These findings indicate that ethylene may also be an important player during fruit set, in parallel to the two well-known players, auxin and GA.

The last step of the ethylene biosynthetic pathway, 1-aminocyclopropane-1-carboxylate oxidase (ACC oxidase) catalyses the conversion of aminocyclopropane-1-carboxylate (ACC) to ethylene (Yang and Hoffman, 1984). During WT fruit set, expression levels of four DEGs encoding ACC oxidase proteins decreased while the other 15 DEGs increased in 2 daa fruitlets compared with 2 dba ovaries (Table 6). Similarly, in the *SlINVINH1*-RNAi plants, four ACC oxidase transcript levels decreased whereas nine transcript levels increased in 2 daa fruitlets compared with 2 dba ovaries (Table 6). The results suggest that ethylene synthesis capacity may be higher in 2 daa fruitlets than 2 dba ovaries in both WT and *SlINVINH1*-RNAi plants. Compared with WT ovaries, four ACC oxidase transcripts exhibited up-regulation with one down-regulated in ovaries of *SlINVINH1*-RNAi plants (Table 6), suggesting that ethylene synthesis may be enhanced in the CWIN-elevated ovaries.

**Table 6.** Numbers of differentially expressed genes encoding 1-aminocyclopropane-1-carboxylate oxidase proteins

Group	Up-regulated	Down-regulated
WT fruitlet *vs* WT ovaries	15	4
*SlINVINH1*-RNAi fruitlet *vs* RNAi ovaries	9	4
*SlINVINH1*-RNAi ovaries *vs* WT ovaries	4	1

In addition to ethylene synthesis genes, expression of a gene encoding an ethylene response factor (ERF) was up-regulated 2.83-fold in *SlINVINH1*-RNAi ovaries compared with WT ovaries (Table 5). Transcripts encoding ERF3 and ERF2a were also up-regulated by 2.26- and 0.78-fold, respectively, in 2 dba ovaries of *SlINVINH1*-RNAi compared with those of WT plants. These two ERFs are annotated as pathogenesis-related (PR) transcriptional factors (Table 5), which belong to the APETALA2/ethylene-responsive element binding protein family, and have been demonstrated to be able to bind to the promoters of several PR genes (Singh *et al.*, 2002). The increased expression of most transcripts encoding ACC oxidase proteins and up-regulation of three *ERFs* imply that ethylene production and sensitivity to ethylene are most likely higher in 2 dba ovaries of *SlINVINH1*-RNAi compared with those of WT plants.

### Defence related R genes were up-regulated in ovaries of *SlINVINH1*-RNAi plants in response to elevated CWIN activity

CWIN is involved in stress responses, including biotic stress (Ruan, 2014) and abiotic stress (McLaughlin and Boyer, 2004; Liu *et al.*, 2013). During biotic stress, R genes encoding proteins containing NBS-IRR domains are often expressed to interact with pathogen effector proteins. Plants have evolved hundreds of R genes to defend themselves against pathogens by activating defence response, including PR gene expression (Lee and Yeom, 2015). In this section, we focus on the expression of stress-related genes, R and PR gene, during fruit set and their response to elevated CWIN activity.

Five out of six DEGs encoding R proteins were up-regulated in 2 dba ovaries of *SlINVINH1*-RNAi compared with those of WT plants (Table 5). Among them, three R genes contain Toll/interleukin-1 receptor (TIR) motif with one R gene containing the coiled-coil (CC) motif in the N-terminal region; the other two R genes may lack the N-terminal motif (Table 5). Apart from the R genes, expression of two genes encoding endochitinase were altered in 2 dba ovaries of *SlINVINH1*-RNAi compared with WT plants (Table 5). Endochitinase belongs to the PR protein family (Ebrahim *et al.*, 2011). Together, elevated CWIN activity not only enhanced expression of R genes but also altered the expression of a PR gene in 2 dba ovaries of *SlINVINH1*-RNAi plants compared with those of WT plants.

### Cell wall-related genes in ovaries of *SlINVINH1*-RNAi plants in response to elevated CWIN activity

Cell wall metabolism has been well studied in the later stages of fruit development, namely, ripening and senescence (Brummell *et al.*, 1999), but not in the early fruit set stage. Cell walls not only provide mechanical support to plants but also function as protective barriers against pathogen attack and a pathway for apoplasmic transport of resources including water, ions, and sugars (Palmer *et al.*, 2015).

There were 13 DEGs encoding proteins involved in cell wall metabolism in 2 dba ovaries of *SlINVINH1*-RNAi compared with WT plants (Table 5), including cellulose synthase, endo-1,4-β-glucanase, β-xylosidase, xyloglucan endotransglucosylase/hydrolase 2, pectin esterase, pectin acetylesteresteras, and expansin (Table 5). The expression changes in these cell wall-related genes indicated that cell wall metabolism was affected in response to elevated CWIN activity in 2 dba ovaries of *SlINVINH1*-RNAi compared with those of WT plants.

### RLK-related genes were largely down-regulated in ovaries of *SlINVINH1*-RNAi plants in response to elevated CWIN activity

Signalling-related genes also were targeted in our analyses, since Glc or Fru produced from CWIN activity in the extracellular space may be sensed by as yet unidentified receptors on the plasma membranes to trigger downstream signal transduction (Ruan, 2014).

There were 13 DEGs encoding RLK proteins, with 11 exhibiting decreased expression levels in 2 dba ovaries of *SlINVINH1*-RNAi compared with those in WT plants. These included two DEGs encoding cytoplasmic RLKs, which also exhibited reduced expression in the transgenic ovaries (Table 5). Unlike other RLKs, which are predicted to localize to the plasma membrane, these two receptor-like cytoplasmic kinases lack an extracellular domain, leading to their cytoplasmic localization (Afzal *et al.*, 2008). Interestingly, expression of a small GTPase gene (Rho of plants; ROP) also was down-regulated in the *SlINVINH1*-RNAi ovaries compared with WT (Table 5).

### Cell cycle genes were generally up-regulated in ovaries of *SlINVINH1*-RNAi plants in response to elevated CWIN activity

Fruit set is characterized by intensive cell division (Ruan, 2012), a process stimulated by Glc signalling that could originate from CWIN activity (Wang and Ruan, 2013). Therefore, we examined expression of cell division-related genes to see how they responded to elevated CWIN activity. We identified two DEGs encoding cyclin B2 in 2 dba ovaries of *SlINVINH1*-RNAi plants (Table 5), which were up-regulated in 2 dba ovaries of *SlINVINH1*-RNAi compared with that in WT plants (Table 5).

### Validation of RNA-seq data by qRT-PCR

To validate the RNA-seq data, we selected six DEGs from Table 5 for qRT-PCR measurements of their respective transcripts from 2 dba ovaries of the RNAi and WT plants. The analyses revealed that all the six genes exhibited the same trends in their RNA fold changes between the two genotypes measured by both methods. We noticed that the fold changes measured by qRT-PCR were generally lower than that from RNA-seq analyses, probably owing to the differences in the sensitivity and specificity between the two approaches (Griffith *et al.*, 2010). Importantly, the regression coefficient between the two datasets is 0.7144 (see Supplementary Fig. S2), which is within the range of reported correlations between RNA-seq and qRT-PCR results (e.g. Xu *et al.*, 2011). Together, the findings indicate the reliability of our RNA-seq data.

### Primary metabolite profiling during fruit set

To understand how the CWIN-mediated changes in transcript profiling as described above may impact on metabolism during fruit set, we undertook a metabolomic analysis for the same set of samples used for RNAseq. In total, 55 metabolites were measured and these were subdivided into four groups: (i) amino acids; (ii) sugars and sugar alcohols; (iii) organic acids; and (iv) miscellaneous (Table 7). Specifically, 24 amino acids, 16 sugars and sugar alcohols, 12 organic acids and three miscellaneous metabolites were detected.

**Table 7.** *Primary metabolites levels in ovary (2 dba) and fruitlets (2 daa) of the wild type (WT) and* SlINVINH1*-RNAi line* Data are normalized to mean values of the wild type ovaries (2 dba). Values are means±SE of three replicates. Bold values indicate significant differences by Student’s *t*-test (*P*<0.01) of the WT fruitlets (2 daa) and *SlINVINH1*-RNAi line compared with the WT ovary (2 dba). Asterisk indicates significant differences by Student’s *t*-test (*P*<0.01) of the *SlINVINH1*-RNAi line fruitlets (2 daa) in comparison with the same line at ovary stage (2 dba).

Metabolite	WT ovaries (2 dba)	WT fruitlets (2 daa)	*SlINVINH1*-RNAi ovaries (2 dba)	*SlINVINH1*-RNAi fruitlets (2 daa)
Amino acids				
Alanine	1 ± 0.14	1.40 ± 0.19	0.84 ± 0.15	0.98 ± 0.03
Alanine, β	1 ± 0.10	1.02 ± 0.00	0.97 ± 0.19	0.88 ± 0.185
Aspartic acid	1 ± 0.07	1.00 ± 0.22	1.03 ± 0.20	0.74 ± 0.03
Arginine	1 ± 0.05	0.98 ± 0.05	0.95 ± 0.05	0.95 ± 0.11
Asparagine	1 ± 0.13	0.92 ± 0.09	1.17 ± 0.10	1.02 ± 0.12*
GABA	1 ± 0.11	1.31 ± 0.16	0.87 ± 0.05	1.22 ± 0.15
Glutamine	1 ± 0.05	1.02 ± 0.08	0.94 ± 0.05	1.05 ± 0.04*
Glutamic acid	1 ± 0.04	1.02 ± 0.06	0.95 ± 0.04	0.96 ± 0.04
Glycine	1 ± 0.15	1.10 ± 0.06	0.71 ± 0.03	0.84 ± 0.02*
Histidine	1 ± 0.13	**1.74 ± 0.02**	0.87 ± 0.01	1.29 ± 0.16*
Isoleucine	1 ± 0.04	0.97 ± 0.11	**0.65 ± 0.09**	0.79 ± 0.11
Lysine	1 ± 0.11	1.15 ± 0.09	0.82 ± 0.09	0.96 ± 0.08
Methionine	1 ± 0.24	**2.40 ± 0.16**	0.79 ± 0.13	1.87 ± 0.23*
Ornithine	1 ± 0.13	1.30 ± 0.06	0.70 ± 0.01	0.99 ± 0.04*
Phenylalanine	1 ± 0.16	**1.60 ± 0.16**	0.64 ± 0.05	1.45 ± 0.14*
Proline	1 ± 0.22	**2.46 ± 0.31**	0.70 ± 0.11	2.60 ± 0.10*
Proline, 4-hydroxy	1 ± 0.08	**3.11 ± 0.12**	0.96 ± 0.06	3.02 ± 0.18*
Pyroglutamic acid	1 ± 0.08	1.03 ± 0.04	0.99 ± 0.02	0.91 ± 0.04*
Serine	1 ± 0.10	1.01 ± 0.04	0.84 ± 0.07	**0.77 ± 0.03**
Threonine	1 ± 0.14	1.09 ± 0.08	0.79 ± 0.11	0.80 ± 0.09
Tryptophan	1 ± 0.08	**0.52 ± 0.03**	**0.74 ± 0.05**	0.39 ± 0.03*
Tyramine	1 ± 0.08	0.85 ± 0.03	0.96 ± 0.03	1.13 ± 0.02*
Tyrosine	1 ± 0.14	**1.49 ± 0.05**	0.89 ± 0.06	1.40 ± 0.05*
Valine	1 ± 0.07	1.40 ± 0.16	0.70 ± 0.12	1.11 ± 0.15
Sugars and sugar alcohols				
Fructose	1 ± 0.03	**0.52 ± 0.05**	**0.68 ± 0.04**	0.63 ± 0.06
Fructose-6-P	1 ± 0.10	**1.97 ± 0.07**	0.91 ± 0.05	1.80 ± 0.14*
Fucose	1 ± 0.04	**0.38 ± 0.03**	**0.67 ± 0.08**	0.36 ± 0.01*
Glucose	1 ± 0.08	0.88 ± 0.14	0.88 ± 0.02	0.78 ± 0.04*
Glucose-6-P	1 ± 0.05	**1.70 ± 0.11**	0.95 ± 0.04	1.39 ± 0.11*
Glycerol	1 ± 0.13	0.80 ± 0.05	0.89 ± 0.05	0.69 ± 0.07*
Inositol, *myo*-	1 ± 0.03	0.95 ± 0.04	0.99 ± 0.03	0.90 ± 0.02*
Isomaltose	1 ± 0.05	1.10 ± 0.02	**0.61 ± 0.06**	**0.54 ± 0.10**
Maltitol	1 ± 0.03	0.81 ± 0.13	0.85 ± 0.06	0.98 ± 0.17
Maltose	1 ± 0.01	**1.44 ± 0.04**	1.02 ± 0.01	1.17 ± 0.06*
Maltotriose	1 ± 0.21	**6.17 ± 0.81**	1.02 ± 0.19	2.83 ± 0.44*
Sucrose	1 ± 0.01	1.04 ± 0.03	0.95 ± 0.07	1.06 ± 0.04
Trehalose	1 ± 0.03	1.22 ± 0.10	0.93 ± 0.04	1.02 ± 0.15
Raffinose	1 ± 0.06	1.13 ± 0.19	1.06 ± 0.07	1.31 ± 0.05*
Rhamnose	1 ± 0.05	0.97 ± 0.09	0.93 ± 0.02	0.92 ± 0.05
Galactinol	1 ± 0.06	0.88 ± 0.01	0.93 ± 0.01	0.90 ± 0.06
Organic acids				
Citric acid	1 ± 0.03	1.04 ± 0.01	0.98 ± 0.04	**0.98 ± 0.01**
Fumaric acid	1 ± 0.03	0.85 ± 0.07	0.89 ± 0.08	0.91 ± 0.08
Galactonic acid	1 ± 0.15	1.03 ± 0.04	0.71 ± 0.07	**0.80 ± 0.07**
Glucuronic acid	1 ± 0.11	**2.18 ± 0.17**	**1.57 ± 0.07**	1.13 ± 0.09*
Glyceric acid	1 ± 0.18	0.72 ± 0.06	0.65 ± 0.05	0.72 ± 0.14
Nicotinic acid	1 ± 0.08	**1.90 ± 0.07**	0.79 ± 0.06	1.92 ± 0.19*
Malic acid	1 ± 0.07	**1.34 ± 0.04**	1.22 ± 0.10	1.24 ± 0.09
Pyruvic acid	1 ± 0.09	**1.38 ± 0.02**	1.34 ± 0.19	1.53 ± 0.12
Quinic acid	1 ± 0.13	0.92 ± 0.08	1.31 ± 0.141	0.95 ± 0.19
Quinic acid, 3-caffeoyl-, *cis*-	1 ± 0.30	**6.21 ± 0.84**	1.22 ± 0.32	6.04 ± 0.88*
Quinic acid, 3-caffeoyl-, *trans*-	1 ± 0.02	0.98 ± 0.01	1.00 ± 0.04	0.95 ± 0.03
Succinic acid	1 ± 0.14	**0.42 ± 0.08**	**0.55 ± 0.01**	0.42 ± 0.03*
Miscellaneous				
Phosphoric acid	1 ± 0.05	1.07 ± 0.01	1.05 ± 0.04	1.09 ± 0.04
Nicotinamide	1 ± 0.05	**1.50 ± 0.09**	**0.61 ± 0.03**	1.13 ± 0.11*
Putrescine	1 ± 0.15	0.60 ± 0.03	0.76 ± 0.02	0.65 ± 0.04*

Among the 24 amino acids, 22 exhibited slightly reduced levels in ovaries of the RNAi plants compared with those in the WT, although only two, isoleucine and tryptophan, showed a statistically significant decrease (Table 7). During the ovary-to-fruitlet transition, six amino acids were significantly increased in WT plants with a similar trend observed for the transgenic plants. Noticeably, levels of methionine, the precursor for ethylene biosynthesis, were doubled during the ovary-to-fruit transition in both genotypes (Table 7).

For the sugar and sugar alcohol group, most members did not show significant changes in the RNAi ovaries compared with those of the WT, except for fructose, fucose, and isomaltulose, which exhibited significant reductions (Table 7). Interestingly, both G-6-P and F-6-P exhibited significantly increased levels across the ovary-to-fruit transition in both WT and *SlINVINH1*-RNAi plants but without being effected by elevated CWIN activity in the transgenic ovaries (Table 7).

Other metabolites exhibiting significantly increased levels in both genotypes during the ovary-to-fruit transition included maltotriose, succinic acid, nicotinamide, nicotinic acid, and glucuronic acid. Among these metabolites it is interesting to note that the levels of glucuronic acid, succinic acid, and nicotinamide were significantly increased in the CWIN-elevated transgenic ovaries and fruitlets as compared with their respective WT counterparts (Table 7).

### Principal component analyses of transcriptome and metabolome data

**Fig. 5.**
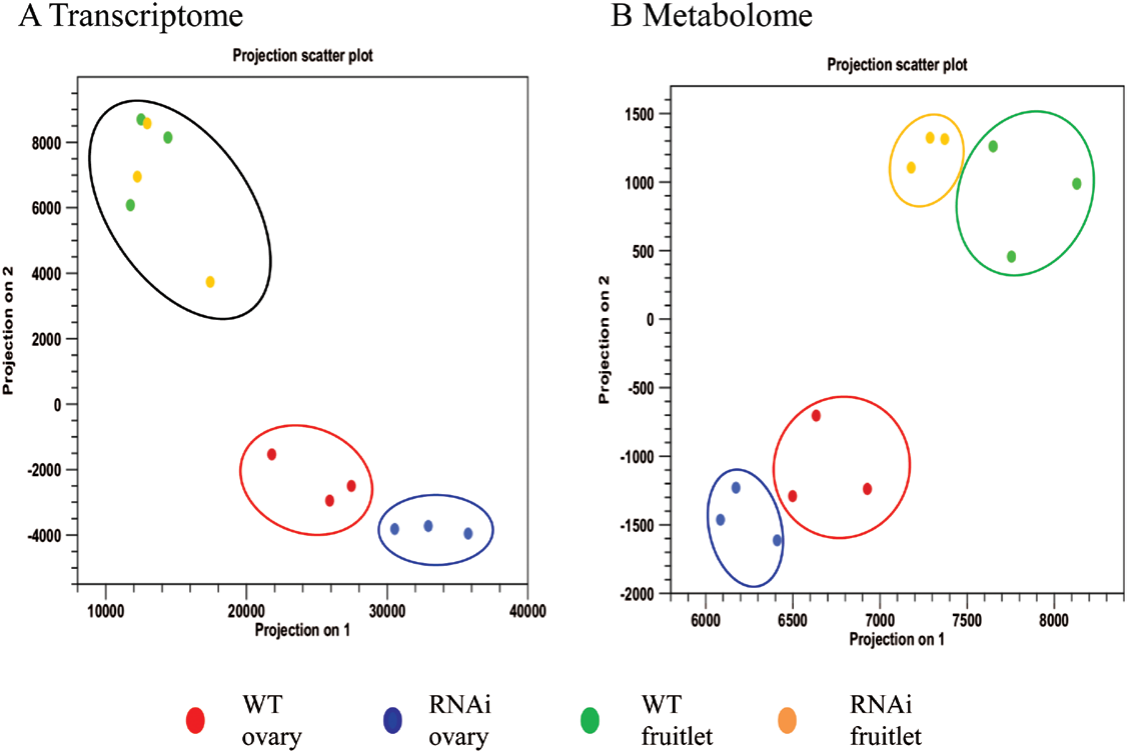
Principal component analyses (PCA) of transcripts and metabolites during fruit set. (A) PCA of the transcriptome data. Two developmental stages were well separated. Discrimination of WT from *SlINVINH1*-RNAi ovaries was detected. However, differences between two genotypes at the fruitlet stage could not be distinguished. (B) PCA of the primary metabolite data. The two developmental stages and two genotypes were both well separated. Three biological replicates were included in both transcriptome and metabolome analyses. (This figure is available in colour at *JXB* online.)

Principal component analyses (PCA) of transcriptome and metabolome data allowed clear visualization of differences between developmental stages of fruit set and genotypes studied. Each point represented the transcriptome or metabolome of the different samples, in a two-dimensional plot. At the ovary stage, where we observed significant transcriptomic shifts in response to elevation of CWIN (Fig. 3), both transcriptome and metabolome were clearly separated between the CWIN-elevated and WT plants (Table 7). At the fruitlet stage (2 daa), however, transcriptome differences could not be distinguished between the two genotypes. By contrast, they separated well into two distinct clusters using the metabolomics data (Fig. 5). We attribute this finding to differences in CWIN activity being more discriminative at the metabolomic compared with the transcriptomic level and to metabolic changes preceding changes at the transcriptional level in the fruitlets. A similar phenomenon was also observed in the study of Wang *et al.* (2009) who compared auxin response factor IAA9 in transgenic and WT tomato plants and found that the difference in the metabolomic data is more discriminative than that in the transcriptomic data in their comparisons.

## Discussion

### Ovaries are transcriptionally far more sensitive to elevated CWIN activity than fruitlets: ready to set

Upon fertilization, tomato ovaries develop into fruitlets. However, the molecular pathways responsible for ovary-to-fruit transition and how they respond to metabolic or biochemical changes remain largely unknown. Our analyses revealed that only seven transcripts exhibited changed levels in response to elevated CWIN activity at the fruitlet stage. By contrast, 319 transcripts were differentially expressed in ovaries of *SlINVINH1*-RNAi (CWIN activity elevated) compared with WT ovaries (Fig. 3), a 45-fold increase in the number of DEGs compared with the fruitlet stage. These findings indicate that ovaries undergo a much more complex regulation in response to elevated CWIN activity at the mRNA level than fruitlets, in order to ensure successful pollination and fertilization.

To the best of our knowledge, this is the first report illustrating that ovaries were much more responsive to elevated CWIN activity compared with fruitlets. Indeed, there are no studies reporting that the ovary is more sensitive to changes in gene expression compared with fruitlets. With this information, we focused on the ovary stage for detailed comparative analyses. In broad terms, this finding is in agreement with our ‘Ready–Set–Growth’ model of fruit set, in which significant molecular and biochemical changes are proposed to take place in the ovary preceding transition to the fruitlet stage upon fertilization (Ruan *et al.*, 2012; Palmer *et al.*, 2015). Consistently, a recent study showed that a group of CWIN and VIN genes in maternal reproductive tissues of tobacco flowers were induced by pollination (Goetz *et al.*, 2017).

### Elevation of CWIN activity increased the expression of genes for defence against pathogens

During fruit set in WT plants, expression levels of most genes encoding R proteins involved in pathogen resistance were higher in 2 dba ovaries than that in 2 daa fruitlets (see Supplementary Table S2). Vriezen *et al.*, (2008) found that expression of genes encoding defence-related proteins decreased from 3 dba ovaries to 3 daa fruitlets in tomato. Our observation, together with that reported by (Vriezen *et al.*, 2008), indicates that ovaries are likely to be under a default active state against potential pathogen attack, probably as a mechanism to protect the critical transition from ovules to seeds and ovaries to fruitlets. The higher expression levels of R genes in 2 dba ovaries compared with 2 daa fruitlets in WT plants (Table 5) also suggests that ovaries are more prone to pathogen attack than fruitlets. Indeed, ovaries are often under attack by many pathogenic fungi, some of which can directly penetrate ovary walls (Ngugi and Scherm, 2006). In addition, tomato fruit can be infected by pathogens such as *Xanthomonas campestris* pv. *vesicatoria*, which causes a serious disease (Oldroyd and Staskawicz, 1998). The R gene-mediated pathway could contribute a broad spectrum of resistance, including resistance against *X. c. vesicatoria* (Oldroyd and Staskawicz, 1998).

Six R genes and two PR genes were responsive to elevated CWIN activity in 2 dba ovaries of *SlINVINH1*-RNAi in comparison with those in WT plants (Table 5). Among them, five R genes exhibited up-regulated expression in transgenic ovaries with one PR gene up- and one down-regulated. These findings indicated that elevation of CWIN activity enhanced overall R gene expression in ovaries, and hence the potential of the transgenic ovaries to defend against pathogen attack. PR gene expression is part of the R gene-mediated defence reaction in plants (Lee and Yeom, 2015). Enhanced PR gene expression by expression of invertases has been reported previously (Herbers *et al.*, 1996; Sonnewald *et al.*, 2012; Sun *et al.*, 2014).

How CWIN activity induces expression of PR genes remains unclear. In this context, transgenic tobacco plants expressing a yeast-derived CWIN or VIN gene developed necrotic lesions in source leaves, typical of a hypersensitive responses (HR) symptom, with three transcripts (*PR-Q*, *PAR1*, *PR-1b*) encoding PR proteins accumulated in the transgenic tobacco leaves (Herbers *et al.*, 1996, 2000). Similar observations have been made recently in source leaves of pepper (Sonnewald *et al.*, 2012) and rice (Sun *et al.*, 2014). These authors proposed that invertase-mediated sugars induced PR gene expression via the secretory pathway through which CWIN and VIN proteins are targeted to the cell wall matrix and vacuoles, respectively. Indeed, CWINs are also considered as PR proteins in their own right (Roitsch *et al.*, 2003). These studies, however, did not report induction of R genes by increased CWIN activity (von Schaewen *et al.*, 1990; Herbers *et al.*, 1996). Our data indicate that the CWIN induces or enhances R gene expression, leading to activation of PR genes.

There are a few partners in the R-gene-mediated pathway for resistance to pathogen infection. One of them is cytoplasmic serine/threonine kinase. In addition to the increased R gene expression in 2 dba ovaries of *SlINVINH1*-RNAi compared with WT ovaries (Table 5), we also found two DEGs encoding cytoplasmic serine/threonine kinases in the *SlINVINH1*-RNAi ovaries (Table 5). A typical example of the interaction of cytoplasmic serine/threonine kinase and R protein is the Prf–Pto complex in tomato pathogen recognition (Ntoukakis *et al.*, 2014). *Prf* is an *R* gene, containing a leucine-rich repeat and nucleotide-binding site motif (Salmeron *et al.*, 1996), with the encoded R protein localized to the cytoplasm (Gururani *et al.*, 2012). *Pto* encodes serine/threonine protein kinase without an extracellular domain, also located in the cytoplasm (Martin *et al.*, 1993; Salmeron *et al.*, 1996). Pto can bind directly to the N-terminus of Prf *in vivo* (Mucyn *et al.*, 2006). However, the exact mechanism of how the Prf–Pto complex works in recognizing effectors secreted by pathogens is unknown (Oh and Martin, 2011). Identification of two DEGs encoding cytoplasmic serine/threonine kinases in the CWIN-elevated tomato ovaries provided an opportunity to examine how alterations in Suc catabolism and signalling may modulate their interaction with R proteins to respond to pathogen infection in tomato.

ERFs, such as Pto-interacting 4 (pti4), are regarded as another partner of the Prf–Pto complex (Wu *et al.*, 2015). An ERF physically interacts with Pto and activates PR gene expression (Zhou *et al.*, 1997; Chakravarthy *et al.*, 2003). The two ERFs in our study were annotated as PR transcriptional factors, and thus may represent other partners in the R-gene mediated pathway.

ROP, a small GTP-binding protein, is also considered to be involved in interaction with the Prf–Pto complex and has a role in ROS production, leading to the HR reaction (Kawano *et al.*, 2010). Interestingly, a ROP gene was down-regulated in *SlINVINH1*-RNAi compared with WT ovaries (Table 5). Apart from its role in HR response, ROP functions as a signalling switch that controls a wide variety of cellular functions including cell division, cell differentiation, cell morphogenesis (Yang, 2002; Miyawaki and Yang, 2014), and plant defence responses (Agrawal *et al.*, 2003; van Zanten *et al.*, 2009; Shpak, 2013). The down-regulation of ROP gene expression in tomato ovaries in response to elevated CWIN activity indicates that CWIN may regulate fruit set by affecting a broad array of cellular development pathways.

Together, the above findings provide opportunities to further study the molecular mechanisms by which CWIN-mediated sugar signalling regulates pathogen resistance in ovaries and fruits through the R-gene mediated pathway, which may involve the expression and function of cytoplasmic serine/threonine kinase, PR ERFs and ROP.

Attention was also given to the possible trade-off for nutrients and energy between plant defence and development (Messina *et al.*, 2002; Huot *et al.*, 2014). Here, levels of most amino acids displayed a slight reduction in 2 dba ovaries of the CWIN-elevated plants compared with those of WT with the difference being statistically significant for isoleucine and tryptophan (Table 7). The reduced size of the free amino acid pool may be derived from decreased expression of protein degradation genes in the transgenic ovaries (Table 5). Alternatively, it may be indicative of enhanced protein synthesis including that for the R proteins since amino acids are building blocks for protein synthesis. The protein content was indeed increased in 10-day-old seeds of *SlINVINH1*-RNAi plants compared with WT plants (Jin *et al.*, 2009). Protein synthesis consumes substantial metabolic energy derived from Glc, which might have impacted fruit development. However, fruit set was not affected under optimal condition and was enhanced under heat stress in *SlINVINH1*-RNAi plants compared with WT plants (Liu *et al.*, 2016), indicating that the energy status was not adversely affected in transgenic ovaries or fruitlets. To this end, expression of most protein degradation-related genes were down-regulated in 2 dba ovaries of *SlINVINH1*-RNA compared with WT ovaries (Table 5). This indicates a prolonged protein turnover rate, which would save metabolic energy for synthesis of new proteins including those for defence-related functions in the CWIN-elevated ovaries to increase their defence capacity for fruit set. Synergistically, enhanced CWIN activity also improved fruit set under long term moderate heat stress (Liu *et al.*, 2016). Thus, elevation of CWIN activity appears to enhance the defence capability of ovaries and fruitlets against both biotic and abiotic stress.

### Elevation of CWIN activity repressed photosynthesis-related genes during ovary-to-fruitlet transition

During ovary-to-fruit transition, we found that the CWIN-elevated plants exhibited decreased transcript levels of a large number of photosynthetic genes controlling both light and dark reactions as compared with the same ovary-to-fruit transition stage in WT plants (Table 2). Consistently, the transgenic fruits displayed a lighter colour late in development (Fig. 2). Repression of photosynthesis genes by expressing a yeast-derived invertase in the apoplast has been reported in mature leaves of tobacco and potato (Heineke *et al.*, 1992). The effect is thought to be caused by a feedback inhibition of Suc export from source leaves (von Schaewen *et al.*, 1990; Dickinson *et al.*, 1991) due to Suc being hydrolysed by CWIN into Glc and Fru, which cannot be loaded into phloem for translocation to sinks organs (von Schaewen *et al.*, 1990). Moreover, high levels of Glc, could inhibit photosynthesis directly though sugar signalling (Jang and Sheen, 1994; Sheen *et al.*, 1999). With higher CWIN activity in the transgenic ovaries and fruitlets, extra Glc is likely to be released from Suc hydrolysis in their apoplasms, which may repress photosynthesis through a feedback system. Physiologically, enhanced assimilate import observed in the RNAi fruit (Liu *et al.*, 2016) may make it unnecessary for fruit to produce extra carbon via photosynthesis. Indeed, a study in which chlorophyll production was down-regulated in tomato revealed that fruit photosynthesis was non-essential for normal fruit function but required for seed set (Lytovchenko *et al.*, 2011).

### Elevation of CWIN activity enhanced expression of ethylene synthesis and cell cycle genes

Genes involved in ethylene synthesis and signalling were ranked third within the hormone-related gene category in terms of number of DEGs after auxin- and GA-related genes expressed during ovary-to-fruit transition (Fig. 4). Previous studies also found that expression of ethylene-related genes was among the most altered across of all hormone-related genes during tomato fruit set (Vriezen *et al.*, 2008; Wang *et al.*, 2009). Auxin and GA are well-recognized hormones during fruit set (Goetz *et al.*, 2007; de Jong *et al.*, 2009). Ethylene may play an antagonistic role to auxin and GA (Vriezen *et al.* (2008). It is suggested to be an activator for fruit set of zucchini squash (Martínez *et al.*, 2013) and tomato (Shinozaki *et al.*, 2015). Studies in orchids revealed that ethylene is also required for ovary development and ovule differentiation together with auxin (Zhang and O’Neill, 1993).

During fruit set, expression of most members of the ethylene synthesis genes, including ACC oxidase, was up-regulated in 2 daa fruitlets compared with 2 dba ovaries in WT and *SlINVINH1*-RNAi plants (Table 6). Consistently, levels of methionine, the precursor of ethylene, more than doubled in fruitlets compared with ovaries in both genotypes (Table 7), indicating positive roles played by ethylene for tomato fruit set. Interestingly, previous transcriptome studies found that mRNA levels of ethylene biosynthesis and signalling genes decreased in 4 daa fruitlets as compared with tomato ovaries at 2 dba (Wang *et al.*, 2009) and at 0 dba (Pattison *et al.*, 2015). These authors compared gene expression profiles of ovaries with 4 daa fruitlets, 2 d older than the 2 daa fruitlets used in our study. The discrepancy in the expression changes of ethylene synthesis and perception genes between our work and those reported by Wang *et al.* (2009) and Pattison *et al.* (2015) likely comes from the different developmental stages at which the samples were harvested and analysed. Recently, ethylene production rate was measured directly by gas chromatography in pistils during tomato fruit set from 2 dba to 4 daa (Shinozaki *et al.*, 2015). However, the pistil is the entire female organ, which could mask the molecular and biochemical status of the ovaries.

In most species, pollination is accompanied by an increase in ethylene production in the stigma and style hours after pollination and there is a burst of ethylene soon after fertilization (O’Neill, 1997; Lin *et al.*, 2009). The up-regulated expression of ethylene synthesis genes during fruit set observed in our study might reflect a short ethylene burst following fertilization. On the other hand, since tomato petals and styles start to senescence soon after fertilization, an increased expression of genes encoding the ACC oxidase enzyme in ovaries (Table 6) may increase ethylene levels, which could induce senescence of petals and styles (Llop-Tous *et al.*, 2000).

In the 2 dba ovaries, we found that four of five genes encoding ACC oxidase increased their mRNA levels in CWIN-elevated plants (Table 5). The up-regulated expression of these genes by increased CWIN activity may contribute to ovule and ovary development in tomato. Consistent with this proposition, silencing ACC oxidase inhibited tobacco ovule development (De Martinis and Mariani, 1999) and enhanced expression of *ACC* genes has been observed in ovaries during their developmental progression to anthesis in tomato (Vriezen *et al.*, 2008).

It is also noteworthy that expression of two *cyclin B2* genes were up-regulated in 2 dba ovaries of *SlINVINH1*- RNAi compared with WT ovaries (Table 5). CWIN-mediated release of hexose plays a role in cell division in seed and fruit development (Cheng and Chourey, 1999; Ruan, 2012, 2014). The B-type cyclins are mitotic cyclins that mediate progression of cells into and out of mitosis (Inzé and De Veylder, 2006). Overexpression of *cycb2;2* in rice promoted root growth by increasing cell number (Lee *et al.*, 2003). *Cycb2;2* has been recently reported to function in establishing rice grain size and yield (Ishimaru *et al.*, 2013) and cell wall formation in mitotic cells of maize endosperm (Sabelli *et al.*, 2014). Thus, up-regulated expression of two *cyclinB* genes in tomato ovaries by elevated CWIN activity may stimulate the cell cycle during fruit set.

### A model of CWIN mediating fruit set

**Fig. 6.**
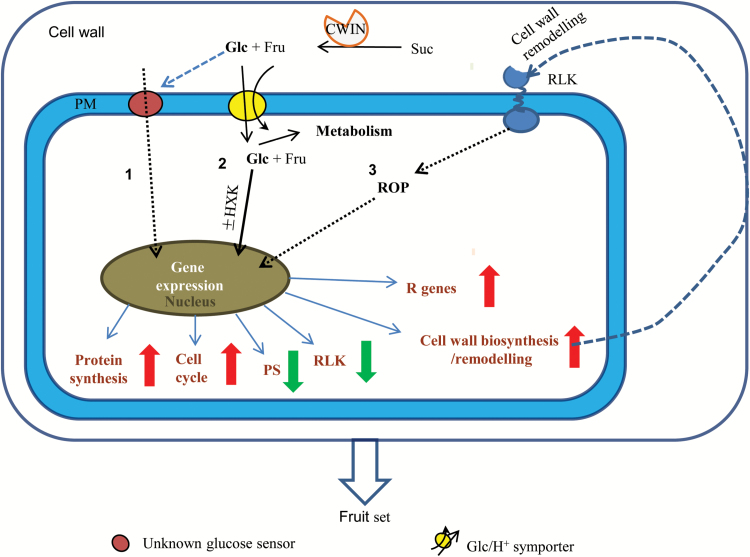
A model of how CWIN-mediated sugar signalling may regulate fruit set. See Discussion for more details. Arrows indicate genes involved in the specified process that were up-regulated (red) or down-regulated (green) by elevated CWIN activity. Solid line indicates known pathway; dashed line indicates predicted pathways.

Based on the above analyses, we propose a model for how CWIN may regulate fruit set (Fig. 6). Here the Glc and Fru produced from CWIN activity in the extracellular space could be (i) sensed by an unknown Glc sensor located on the plasma membrane (PM) to regulate gene expression and (ii) be taken up by the hexose transporter into the cytosol for metabolism or sugar signalling (Ruan *et al.*, 1995, 2012; Ruan 2014). Elevation of CWIN activity up-regulates expression of genes encoding proteins involved in defense (R genes), protein synthesis, cell cycle, and cell wall biosynthesis/remodelling, but down-regulates photosynthesis genes and receptor-like kinase (RLK) in ovaries of *SlINVINH1*-RNAi compared with WT plants. The genes encoding proteins functioning in cell wall biosynthesis/remodelling may cause changes in cell wall components, which may be sensed by RLK located on the plasma membrane to regulate gene expression through a small GTPase (ROP) pathway. Together, the model provides insights into the molecular pathways by which CWIN positively regulates fruit set.

## Supplementary data

Supplementary data are available at *JXB* online.

Fig. S1. Invertase activities in 2 dba ovaries and 2 daa fruitlets in the *SlINVINH1*-RNAi plants as compared with the WT.

Fig. S2. Validation of RNA-seq data by qRT-PCR.

Table S1. Primers used for qRT-PCR analyses.

Table S2. A total of 5183 differentially expressed genes in wild type at fruit set

Table S3. A total of 3262 differentially expressed genes during fruit set of *SlINVINH1*-RNAi plants.

Table S4. A total of 497 differentially expressed genes in *SlINVINH1*-RNAi fruit set in response to elevated CWIN activity compared with wild type fruit set.

Table S5. A total of 319 differentially expressed genes in ovaries of *SlINVINH1*-RNAi compared with those of wild type plants.

## Supplementary Material

Supplementary Table S1

Supplementary Table S2

Supplementary Table S3

Supplementary Table S4

Supplementary Figures S1-S2 and Table S5

## References

[CIT0001] Afzal AJ, Wood AJ, Lightfoot DA 2008 Plant receptor-like serine threonine kinases: roles in signaling and plant defense. Molecular Plant-Microbe Interactions 21, 507–517. 18393610 10.1094/MPMI-21-5-0507

[CIT0002] Agrawal GK, Iwahashi H, Rakwal R 2003 Small GTPase ‘Rop’: molecular switch for plant defense responses. FEBS Letters 546, 173–180. 12832035 10.1016/s0014-5793(03)00646-x

[CIT0003] Alba R, Payton P, Fei Z, McQuinn R, Debbie P, Martin GB, Tanksley SD, Giovannoni JJ 2005 Transcriptome and selected metabolite analyses reveal multiple points of ethylene control during tomato fruit development. The Plant Cell 17, 2954–2965. 16243903 10.1105/tpc.105.036053 PMC1276022

[CIT0004] Beauvoit BP, Colombié S, Monier A 2014 Model-assisted analysis of sugar metabolism throughout tomato fruit development reveals enzyme and carrier properties in relation to vacuole expansion. The Plant Cell 26, 3224–3242. 25139005 10.1105/tpc.114.127761 PMC4371827

[CIT0005] Brummell DA, Harpster MH, Civello PM, Palys JM, Bennett AB, Dunsmuir P 1999 Modification of expansin protein abundance in tomato fruit alters softening and cell wall polymer metabolism during ripening. The Plant Cell 11, 2203–2216. 10559444 10.1105/tpc.11.11.2203 PMC144123

[CIT0006] Carrari F, Fernie AR 2006 Metabolic regulation underlying tomato fruit development. Journal of Experimental Botany 57, 1883–1897. 16449380 10.1093/jxb/erj020

[CIT0007] Chakravarthy S, Tuori RP, D’Ascenzo MD, Fobert PR, Despres C, Martin GB 2003 The tomato transcription factor Pti4 regulates defense-related gene expression via GCC box and non-GCC box cis elements. The Plant Cell 15, 3033–3050. 14630974 10.1105/tpc.017574 PMC282854

[CIT0008] Cheng WH, Chourey PS 1999 Genetic evidence that invertase-mediated release of hexoses is critical for appropriate carbon partitioning and normal seed development in maize. Theoretical and Applied Genetics 98, 485–495.

[CIT0009] Cheng WH, Taliercio EW, Chourey PS 1996 The Miniature1 seed locus of maize encodes a cell wall invertase required for normal development of endosperm and maternal cells in the pedicel. The Plant Cell 8, 971–983. 12239408 10.1105/tpc.8.6.971 PMC161152

[CIT0010] de Jong M, Mariani C, Vriezen WH 2009 The role of auxin and gibberellin in tomato fruit set. Journal of Experimental Botany 60, 1523–1532. 19321650 10.1093/jxb/erp094

[CIT0011] De Martinis D, Mariani C 1999 Silencing gene expression of the ethylene-forming enzyme results in a reversible inhibition of ovule development in transgenic tobacco plants. The Plant Cell 11, 1061–1072. 10368177 10.1105/tpc.11.6.1061 PMC144249

[CIT0012] Dickinson CD, Altabella T, Chrispeels MJ 1991 Slow-growth phenotype of transgenic tomato expressing apoplastic invertase. Plant Physiology 95, 420–425. 16668000 10.1104/pp.95.2.420 PMC1077547

[CIT0013] Ebrahim S, Usha K, Singh B 2011 Pathogenesis related (PR) proteins in plant defense mechnism. In: Méndez-Vilas A, ed. Science against microbial pathogens: communicating current research and technological advance. Badajoz: Formatex, 1043–1054.

[CIT0014] Goetz M, Guivarćh A, Hirsche J 2017 Metabolic control of tobacco pollination by sugars and invertases. Plant Physiology 173, 984–997. 27923989 10.1104/pp.16.01601 PMC5291038

[CIT0015] Goetz M, Hooper LC, Johnson SD, Rodrigues JC, Vivian-Smith A, Koltunow AM 2007 Expression of aberrant forms of *AUXIN RESPONSE FACTOR8* stimulates parthenocarpy in Arabidopsis and tomato. Plant Physiology 145, 351–366. 17766399 10.1104/pp.107.104174 PMC2048734

[CIT0016] Griffith M, Griffith OL, Mwenifumbo J 2010 Alternative expression analysis by RNA sequencing. Nature Methods 7, 843–847. 20835245 10.1038/nmeth.1503

[CIT0017] Gururani MA, Venkatesh J, Upadhyaya CP, Nookaraju A, Pandey SK, Park SW 2012 Plant disease resistance genes: Current status and future directions. Physiological and Molecular Plant Pathology 78, 51–65.

[CIT0018] Heineke D, Sonnewald U, Bussis D, Gunter G, Leidreiter K, Wilke I, Raschke K, Willmitzer L, Heldt HW 1992 Apoplastic expression of yeast-derived invertase in potato. Effects on photosynthesis, leaf solute composition, water relations, and tuber composition. Plant Physiology 100, 301–308. 16652961 10.1104/pp.100.1.301 PMC1075552

[CIT0019] Herbers K, Meuwly P, Frommer WB, Métraux JP, Sonnewalda U 1996 Systemic acquired resistance mediated by the ectopic expression of invertase: possible hexose sensing in the secretory pathway. The Plant Cell 8, 793–803. 12239401 10.1105/tpc.8.5.793 PMC161138

[CIT0020] Herbers K, Takahata Y, Melzer M, Mock HP, Hajirezaei M, Sonnewald U 2000 Regulation of carbohydrate partitioning during the interaction of potato virus Y with tobacco. Molecular Plant Pathology 1, 51–59. 20572950 10.1046/j.1364-3703.2000.00007.x

[CIT0021] Huot B, Yao J, Montgomery BL, He SY 2014 Growth-defense tradeoffs in plants: a balancing act to optimize fitness. Molecular Plant 7, 1267–1287. 24777989 10.1093/mp/ssu049 PMC4168297

[CIT0022] Inzé D, De Veylder L 2006 Cell cycle regulation in plant development. Annual Review of Genetics 40, 77–105. 10.1146/annurev.genet.40.110405.090431 17094738

[CIT0023] Ishimaru K, Hirotsu N, Madoka Y 2013 Loss of function of the IAA-glucose hydrolase gene TGW6 enhances rice grain weight and increases yield. Nature Genetics 45, 707–711. 23583977 10.1038/ng.2612

[CIT0024] Jang JC, Sheen J 1994 Sugar sensing in higher plants. The Plant Cell 6, 1665–1679. 7827498 10.1105/tpc.6.11.1665 PMC160552

[CIT0025] Jin Y, Ni DA, Ruan YL 2009 Posttranslational elevation of cell wall invertase activity by silencing its inhibitor in tomato delays leaf senescence and increases seed weight and fruit hexose level. The Plant Cell 21, 2072–2089. 19574437 10.1105/tpc.108.063719 PMC2729613

[CIT0026] Kawano Y, Chen L, Shimamoto K 2010 The function of Rac small GTPase and associated proteins in rice innate immunity. Rice 3, 112–121.

[CIT0027] Kopka J, Schauer N, Krueger S 2004 GMD@CSB.DB: the Golm Metabolome Database. Bioinformatics 21, 1635–1638. 15613389 10.1093/bioinformatics/bti236

[CIT0028] Kumar R, Khurana A, Sharma AK 2014 Role of plant hormones and their interplay in development and ripening of fleshy fruits. Journal of Experimental Botany 65, 4561–4575. 25028558 10.1093/jxb/eru277

[CIT0029] Lee HA, Yeom SI 2015 Plant NB-LRR proteins: tightly regulated sensors in a complex manner. Briefings in Functional Genomics 14, 233–242. 25825425 10.1093/bfgp/elv012

[CIT0030] Lee J, Das A, Yamaguchi M, Hashimoto J, Tsutsumi N, Uchimiya H, Umeda M 2003 Cell cycle function of a rice B2-type cyclin interacting with a B-type cyclin-dependent kinase. The Plant Journal 34, 417–425. 12753582 10.1046/j.1365-313x.2003.01736.x

[CIT0031] Leon P, Sheen J 2003 Sugar and hormone connections. Trends in Plant Science 8, 110–116. 12663220 10.1016/S1360-1385(03)00011-6

[CIT0032] Lin Z, Zhong S, Grierson D 2009 Recent advances in ethylene research. Journal of Experimental Botany 60, 3311–3336. 19567479 10.1093/jxb/erp204

[CIT0033] Liu YH, Offler CE, Ruan YL 2013 Regulation of fruit and seed response to heat and drought by sugars as nutrients and signals. Frontiers in Plant Science 4, 282. 23914195 10.3389/fpls.2013.00282 PMC3729977

[CIT0034] Liu YH, Offler CE, Ruan YL 2016 Cell wall invertase promotes fruit set under heat stress by suppressing ROS-independent cell death. Plant Physiology 172, 163–180. 27462084 10.1104/pp.16.00959 PMC5074634

[CIT0035] Llop-Tous I, Barry CS, Grierson D 2000 Regulation of ethylene biosynthesis in response to pollination in tomato flowers. Plant Physiology 123, 971–978. 10889245 10.1104/pp.123.3.971 PMC59059

[CIT0036] Lytovchenko A, Eickmeier I, Pons C 2011 Tomato fruit photosynthesis is seemingly unimportant in primary metabolism and ripening but plays a considerable role in seed development. Plant Physiology 157, 1650–1663. 21972266 10.1104/pp.111.186874 PMC3327185

[CIT0037] Martin GB, Brommonschenkel SH, Chunwongse J, Frary A, Ganal MW, Spivey R, Wu T, Earle ED, Tanksley SD 1993 Map-based cloning of a protein kinase gene conferring disease resistance in tomato. Science 262, 1432–1436. 7902614 10.1126/science.7902614

[CIT0038] Martínez C, Manzano S, Megías Z, Garrido D, Picó B, Jamilena M 2013 Involvement of ethylene biosynthesis and signalling in fruit set and early fruit development in zucchini squash (*Cucurbita pepo* L.). BMC Plant Biology 13, 139. 24053311 10.1186/1471-2229-13-139 PMC3856489

[CIT0039] McLaughlin JE, Boyer JS 2004 Sugar-responsive gene expression, invertase activity, and senescence in aborting maize ovaries at low water potentials. Annals of Botany 94, 675–689. 15355866 10.1093/aob/mch193 PMC4242214

[CIT0040] Messina FJ, Durham SL, Richards JH, McArthur DE 2002 Trade-off between plant growth and defense? A comparison of sagebrush populations. Oecologia 131, 43–51. 28547509 10.1007/s00442-001-0859-3

[CIT0041] Miyawaki KN, Yang Z 2014 Extracellular signals and receptor-like kinases regulating ROP GTPases in plants. Frontiers in Plant Science 5, 449. 25295042 10.3389/fpls.2014.00449 PMC4170102

[CIT0042] Mucyn TS, Clemente A, Andriotis VM, Balmuth AL, Oldroyd GE, Staskawicz BJ, Rathjen JP 2006 The tomato NBARC-LRR protein Prf interacts with Pto kinase in vivo to regulate specific plant immunity. The Plant Cell 18, 2792–2806. 17028203 10.1105/tpc.106.044016 PMC1626632

[CIT0043] Ngugi HK, Scherm H 2006 Biology of flower-infecting fungi. Annual Review of Phytopathology 44, 261–282. 10.1146/annurev.phyto.44.070505.143405 17061917

[CIT0044] Ntoukakis V, Saur IM, Conlan B, Rathjen JP 2014 The changing of the guard: the Pto/Prf receptor complex of tomato and pathogen recognition. Current Opinion in Plant Biology 20, 69–74. 24845576 10.1016/j.pbi.2014.04.002

[CIT0045] Oh CS, Martin GB 2011 Effector-triggered immunity mediated by the Pto kinase. Trends in Plant Science 16, 132–140. 21112235 10.1016/j.tplants.2010.11.001

[CIT0046] Oldroyd GE, Staskawicz BJ 1998 Genetically engineered broad-spectrum disease resistance in tomato. Proceedings of the National Academy of Sciences, USA 95, 10300–10305. 10.1073/pnas.95.17.10300 PMC21503 9707642

[CIT0047] O’Neill SD 1997 Pollination regulation of flower development. Annual Review of Plant Physiology and Plant Molecular Biology 48, 547–574. 10.1146/annurev.arplant.48.1.547 15012274

[CIT0048] Osorio S, Do P, Fernie A 2012 Profiling primary metabolites of tomato fruit with gas chromatography/mass spectrometry. Methods in Molecular Biology 860, 101–109. 22351173 10.1007/978-1-61779-594-7_7

[CIT0049] Palmer WM, Ru L, Jin Y, Patrick JW, Ruan YL 2015 Tomato ovary-to-fruit transition is characterized by a spatial shift of mRNAs for cell wall invertase and its inhibitor with the encoded proteins localized to sieve elements. Molecular Plant 8, 315–328. 25680776 10.1016/j.molp.2014.12.019

[CIT0050] Pattison RJ, Csukasi F, Zheng Y, Fei Z, van der Knaap E, Catalá C 2015 Comprehensive tissue-specific transcriptome analysis reveals distinct regulatory programs during early tomato fruit development. Plant Physiology 168, 1684–1701. 26099271 10.1104/pp.15.00287 PMC4528740

[CIT0051] Roitsch T, Balibrea ME, Hofmann M, Proels R, Sinha AK 2003 Extracellular invertase: key metabolic enzyme and PR protein. Journal of Experimental Botany 54, 513–524. 12508062 10.1093/jxb/erg050

[CIT0052] Ruan YL 2012 Signaling role of sucrose metabolism in development. Molecular Plant 5, 763–765. 22532605 10.1093/mp/sss046

[CIT0053] Ruan YL 2014 Sucrose metabolism: gateway to diverse carbon use and sugar signaling. Annual Review of Plant Biology 65, 33–67. 10.1146/annurev-arplant-050213-040251 24579990

[CIT0054] Ruan YL, Mate C, Patrick JW, Brady CJ 1995 Non-destructive collection of apoplast fluid from developing tomato fruit using a pressure dehydration procedure. Functional Plant Biology 22, 761–769.

[CIT0055] Ruan YL, Patrick JW, Bouzayen M, Osorio S, Fernie AR 2012 Molecular regulation of seed and fruit set. Trends in Plant Science 17, 656–665. 22776090 10.1016/j.tplants.2012.06.005

[CIT0056] Sabelli PA, Dante RA, Nguyen HN, Gordon-Kamm WJ, Larkins BA 2014 Expression, regulation and activity of a B2-type cyclin in mitotic and endoreduplicating maize endosperm. Frontiers in Plant Science 5, 561. 25368625 10.3389/fpls.2014.00561 PMC4201103

[CIT0057] Salmeron JM, Oldroyd GE, Rommens CM, Scofield SR, Kim HS, Lavelle DT, Dahlbeck D, Staskawicz BJ 1996 Tomato Prf is a member of the leucine-rich repeat class of plant disease resistance genes and lies embedded within the Pto kinase gene cluster. Cell 86, 123–133. 8689679 10.1016/s0092-8674(00)80083-5

[CIT0058] Sheen J, Zhou L, Jang JC 1999 Sugars as signaling molecules. Current Opinion in Plant Biology 2, 410–418. 10508760 10.1016/s1369-5266(99)00014-x

[CIT0059] Shinozaki Y, Hao S, Kojima M 2015 Ethylene suppresses tomato (*Solanum lycopersicum*) fruit set through modification of gibberellin metabolism. The Plant Journal 83, 237–251. 25996898 10.1111/tpj.12882

[CIT0060] Shpak ED 2013 Diverse roles of ERECTA family genes in plant development. Journal of Integrative Plant Biology 55, 1238–1250. 24016315 10.1111/jipb.12108

[CIT0061] Siezen RJ, Leunissen JA 1997 Subtilases: the superfamily of subtilisin-like serine proteases. Protein Science 6, 501–523. 9070434 10.1002/pro.5560060301 PMC2143677

[CIT0062] Singh K, Foley RC, Oñate-Sánchez L 2002 Transcription factors in plant defense and stress responses. Current Opinion in Plant Biology 5, 430–436. 12183182 10.1016/s1369-5266(02)00289-3

[CIT0063] Sonnewald S, Priller JP, Schuster J, Glickmann E, Hajirezaei MR, Siebig S, Mudgett MB, Sonnewald U 2012 Regulation of cell wall-bound invertase in pepper leaves by *Xanthomonas campestris* pv. *vesicatoria* type three effectors. PLoS ONE 7, e51763. 23272161 10.1371/journal.pone.0051763 PMC3522709

[CIT0064] Sun L, Yang DL, Kong Y, Chen Y, Li XZ, Zeng LJ, Li Q, Wang ET, He ZH 2014 Sugar homeostasis mediated by cell wall invertase GRAIN INCOMPLETE FILLING 1 (GIF1) plays a role in pre-existing and induced defence in rice. Molecular Plant Pathology 15, 161–173. 24118770 10.1111/mpp.12078 PMC6638756

[CIT0065] Thimm O, Bläsing O, Gibon Y, Nagel A, Meyer S, Krüger P, Selbig J, Müller LA, Rhee SY, Stitt M 2004 MAPMAN: a user-driven tool to display genomics data sets onto diagrams of metabolic pathways and other biological processes. The Plant Journal 37, 914–939. 14996223 10.1111/j.1365-313x.2004.02016.x

[CIT0066] van Zanten M, Snoek LB, Proveniers MC, Peeters AJ 2009 The many functions of ERECTA. Trends in Plant Science 14, 214–218. 19303350 10.1016/j.tplants.2009.01.010

[CIT0067] von Schaewen A, Stitt M, Schmidt R, Sonnewald U, Willmitzer L 1990 Expression of a yeast-derived invertase in the cell wall of tobacco and Arabidopsis plants leads to accumulation of carbohydrate and inhibition of photosynthesis and strongly influences growth and phenotype of transgenic tobacco plants. The EMBO Journal 9, 3033–3044. 2209536 10.1002/j.1460-2075.1990.tb07499.x PMC552027

[CIT0068] Vriezen WH, Feron R, Maretto F, Keijman J, Mariani C 2008 Changes in tomato ovary transcriptome demonstrate complex hormonal regulation of fruit set. New Phytologist 177, 60–76. 18028300 10.1111/j.1469-8137.2007.02254.x

[CIT0069] Wang E, Wang J, Zhu X 2008 Control of rice grain-filling and yield by a gene with a potential signature of domestication. Nature Genetics 40, 1370–1374. 18820698 10.1038/ng.220

[CIT0070] Wang H, Schauer N, Usadel B, Frasse P, Zouine M, Hernould M, Latché A, Pech JC, Fernie AR, Bouzayen M 2009 Regulatory features underlying pollination-dependent and -independent tomato fruit set revealed by transcript and primary metabolite profiling. The Plant Cell 21, 1428–1452. 19435935 10.1105/tpc.108.060830 PMC2700536

[CIT0071] Wang L, Ruan YL 2012 New insights into roles of cell wall invertase in early seed development revealed by comprehensive spatial and temporal expression patterns of GhCWIN1 in cotton. Plant Physiology 160, 777–787. 22864582 10.1104/pp.112.203893 PMC3461555

[CIT0072] Wang L, Ruan YL 2013 Regulation of cell division and expansion by sugar and auxin signaling. Frontiers in Plant Science 4, 163. 23755057 10.3389/fpls.2013.00163 PMC3667240

[CIT0073] Wu C, Avila CA, Goggin FL 2015 The ethylene response factor Pti5 contributes to potato aphid resistance in tomato independent of ethylene signalling. Journal of Experimental Botany 66, 559–570. 25504643 10.1093/jxb/eru472 PMC4286409

[CIT0074] Xu L, Zhu L, Tu L, Liu L, Yuan D, Jin L, Long L, Zhang X 2011 Lignin metabolism has a central role in the resistance of cotton to the wilt fungus *Verticillium dahliae* as revealed by RNA-Seq-dependent transcriptional analysis and histochemistry. Journal of Experimental Botany 62, 5607–5621. 21862479 10.1093/jxb/err245 PMC3223054

[CIT0075] Yang SF, Hoffman NE 1984 Ethylene biosynthesis and its regulation in higher plants. Annual Review of Plant Physiology 35, 155–189.

[CIT0076] Yang Z 2002 Small GTPases: versatile signaling switches in plants. The Plant Cell 14, S375–S388. 12045289 10.1105/tpc.001065 PMC151267

[CIT0077] Zanor MI, Osorio S, Nunes-Nesi A 2009 RNA interference of LIN5 in tomato confirms its role in controlling Brix content, uncovers the influence of sugars on the levels of fruit hormones, and demonstrates the importance of sucrose cleavage for normal fruit development and fertility. Plant Physiology 150, 1204–1218. 19439574 10.1104/pp.109.136598 PMC2705052

[CIT0078] Zhang HM, Wheeler S, Xia X, Radchuk R, Weber H, Offler CE, Patrick JW 2015 Differential transcriptional networks associated with key phases of ingrowth wall construction in trans-differentiating epidermal transfer cells of *Vicia faba* cotyledons. BMC Plant Biology 15, 103. 25887034 10.1186/s12870-015-0486-5 PMC4437447

[CIT0079] Zhang XS, O’Neill SD 1993 Ovary and gametophyte development are coordinately regulated by auxin and ethylene following pollination. The Plant Cell 5, 403–418. 12271070 10.1105/tpc.5.4.403 PMC160280

[CIT0080] Zhou J, Tang X, Martin GB 1997 The Pto kinase conferring resistance to tomato bacterial speck disease interacts with proteins that bind a cis-element of pathogenesis-related genes. The EMBO Journal 16, 3207–3218. 9214637 10.1093/emboj/16.11.3207 PMC1169938

