# Supplementary material for: Transcriptomic and metabolomics responses to elevated cell wall invertase activity during tomato fruit set
Source: J Exp Bot. 2017 Jul 20;68(15):4263–79. doi: 10.1093/jxb/erx219 (PMC5853505; doi:10.1093/jxb/erx219)
Supplement: Supplementary Figures S1-S2 and Table S5 [file erx219_suppl_supplementary-figures-s1-s2-table-s5.pdf]

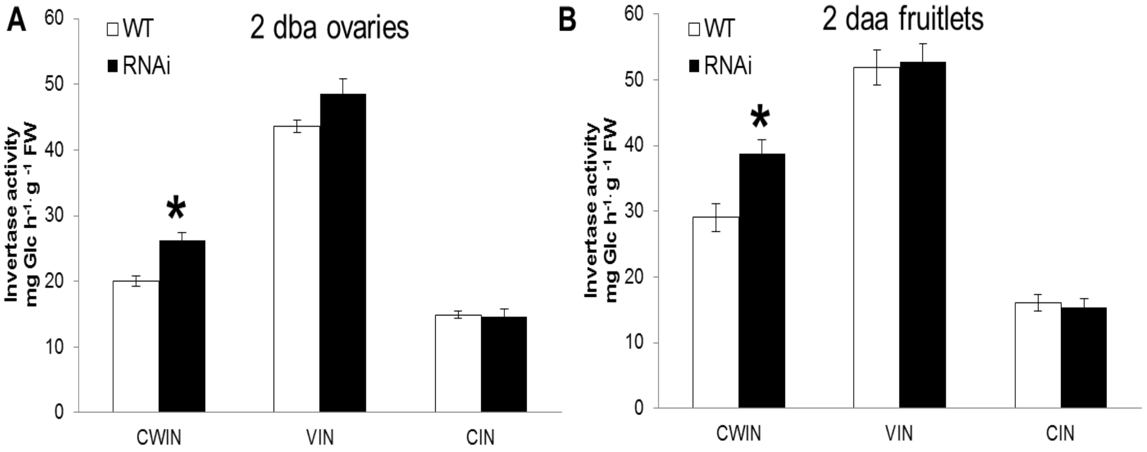

Supplemental Figure 1: Invertase activities in 2 DBA ovaries and 2 BAA fruitlets in the SlINVINH1-RNAi plants as compared that in the WT.
Note a significant increase in activity of cell wall invertase (CWIN) but not that of vacuolar or cytosolic invertases (VIN and CIN, respectively) in the RNAi organs as compared to that of the WT ones. * t test at $p < 0.05$.

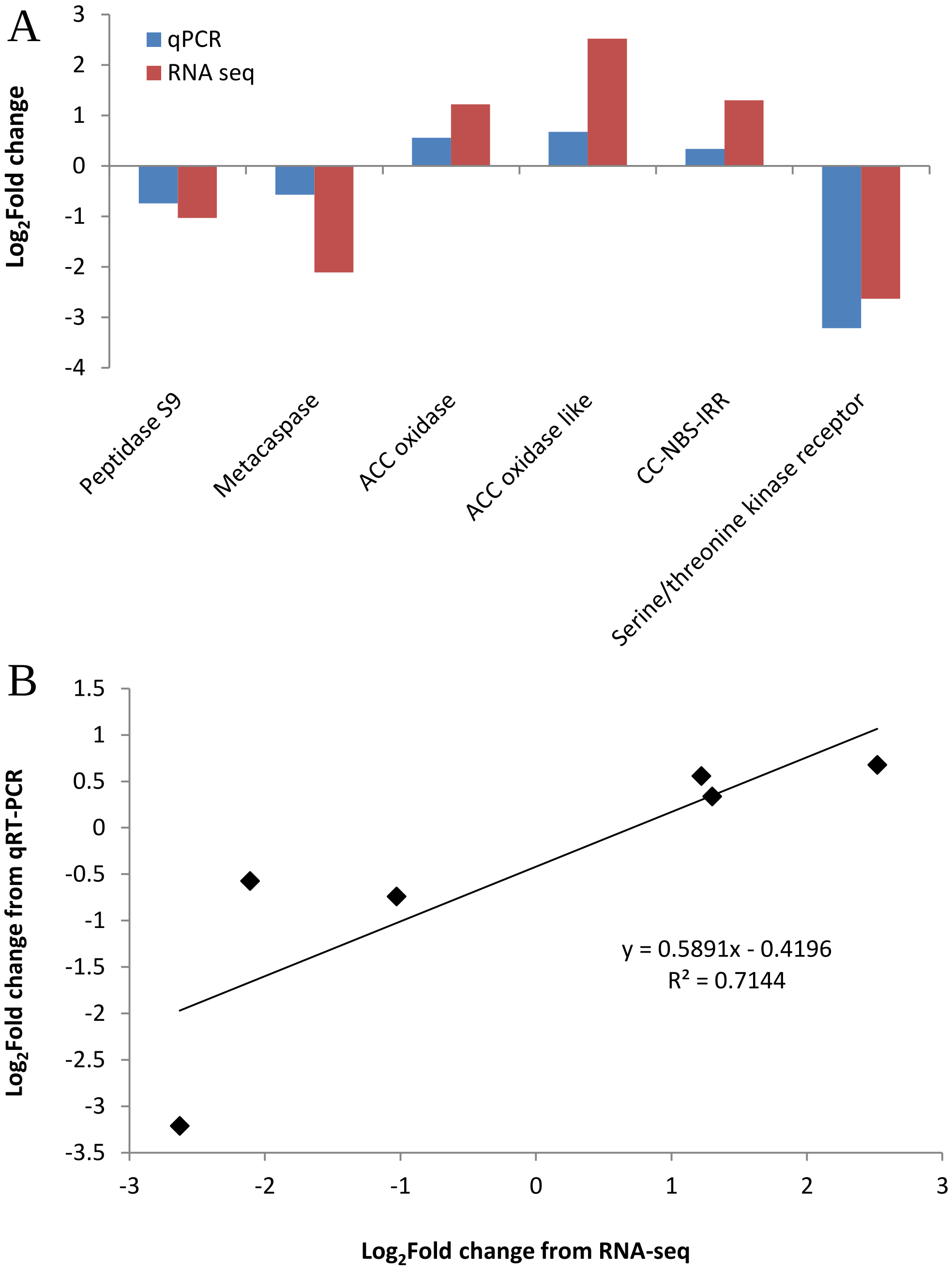

Supplemental Figure 2: Validation of RNA-seq data by qRT-PCR.
(A) Fold changes of transcript levels from 6 genes expressed in 2 DBP ovaries of SlINVINH1 RNAi as compared to that in WT plants measured by RNAseq and qRT-PCR.
(B) Linear regression analysis for the fold changes among the 6 genes for qRT-PCR data and RNA-seq data.

Supplemental Table S5. Primers used for qRT-PCR

Primer Name	Locus ID		Sequence (5'->3')
Peptidase S9	Solyc09g083120.2.1	Forward primer	ATCTCTTCCAGGGAGGGTGG
		Reverse primer	GCAAACTTGTCTGGTGCCTG
Metacaspase	Solyc05g052130.2.1	Forward primer	AACTCCGGCTTTGGGATGAG
		Reverse primer	GAAGGAGCGGCGTAGACTTA
ACC oxidase	Solyc01g067620.2.1	Forward primer	AGGGAAAAAGACTCTTGAGTTTGC
		Reverse primer	GGAACAAAAAGACTAAAGGACACAA
ACC oxidase like	Solyc04g009850.2.1	Forward primer	TAGAGGACTTGGGGCTCACC
		Reverse primer	TCCACGAGTCGAAAACCAAA
CC-NBS-IRR	solyc04g012010.2.1	Forward primer	GGCGTACCGTGTCAAGAGAT
		Reverse primer	ACTGGTAGCTTCCCAATGCT
Serine/threonine kinase receptor	Solyc04g077270.2.1	Forward primer	GGCAGAAGCTAAAGAACCAGG
		Reverse primer	GATGCTCGAGCGCTTCAATG
SlCAC	Solyc08g006960.2	Forward primer	CCTCCGTTGTGATGTAACTGG
		Reverse primer	ATTGGTGGAAAGTAACATCATCG
SlTIP41	Solyc01g107420.2	Forward primer	ATGGAGTTTTTGAGTCTTCTGC
		Reverse primer	GCTGCGTTTCTGGCTTAGG
